# Phenylpropanoid Scent Compounds in *Petunia* x *hybrida* Are Glycosylated and Accumulate in Vacuoles

**DOI:** 10.3389/fpls.2017.01898

**Published:** 2017-11-03

**Authors:** Alon Cna'ani, Reut Shavit, Jasmin Ravid, Javiera Aravena-Calvo, Oded Skaliter, Tania Masci, Alexander Vainstein

**Affiliations:** ^1^Institute of Plant Sciences and Genetics in Agriculture, The Robert H. Smith Faculty of Agriculture, Food and Environment, Hebrew University of Jerusalem, Rehovot, Israel; ^2^Department of Plant Pathology and Microbiology, The Robert H. Smith Faculty of Agriculture, Food and Environment, The Hebrew University of Jerusalem, Rehovot, Israel

**Keywords:** emission, floral scent, glycoside, glycosylation, petunia, phenylpropanoid, vacuole, volatile

## Abstract

Floral scent has been studied extensively in the model plant *Petunia*. However, little is known about the intracellular fate of scent compounds. Here, we characterize the glycosylation of phenylpropanoid scent compounds in *Petunia* x *hybrida*. This modification reduces scent compounds' volatility, reactivity, and autotoxicity while increasing their water-solubility. Gas chromatography–mass spectrometry (GC–MS) analyses revealed that flowers of petunia cultivars accumulate substantial amounts of glycosylated scent compounds and that their increasing level parallels flower development. In contrast to the pool of accumulated aglycones, which drops considerably at the beginning of the light period, the collective pool of glycosides starts to increase at that time and does not decrease thereafter. The glycoside pool is dynamic and is generated or catabolized during peak scent emission, as inferred from phenylalanine isotope-feeding experiments. Using several approaches, we show that phenylpropanoid scent compounds are stored as glycosides in the vacuoles of petal cells: ectopic expression of *Aspergillus niger* β*-glucosidase-1* targeted to the vacuole resulted in decreased glycoside accumulation; GC–MS analysis of intact vacuoles isolated from petal protoplasts revealed the presence of glycosylated scent compounds. Accumulation of glycosides in the vacuoles seems to be a common mechanism for phenylpropanoid metabolites.

## Introduction

Floral scent is a complex trait that, together with pigmentation, nectar production, and flower architecture, enables plants to attract pollinators, thereby allowing sexual reproduction (Hoballah et al., 2007; Klahre et al., 2011; Gaffal, 2012). Scent emitted by flowers is typically a mixture of low-molecular-weight volatile compounds originating from terpenoid, fatty acid, and phenylpropanoid pathways (Muhlemann et al., 2014). Phenylpropanoid volatiles are derived from phenylalanine (Phe), a product of the shikimate pathway, and are further divided into three subclasses: C6–C1 carbon backbone benzenoids, C6–C2 phenylpropanoid-related compounds, and C6–C3 phenylpropenes (Muhlemann et al., 2014). In recent years, numerous genes encoding structural enzymes in the phenylpropanoid pathway, as well as factors regulating metabolic flow within the pathway, have been isolated and characterized (Kaminaga et al., 2006; Moerkercke et al., 2009; Spitzer-Rimon et al., 2010; Klempien et al., 2012; Van Moerkercke et al., 2012; Pan et al., 2014; Fenske et al., 2015; Widhalm et al., 2015; Adebesin et al., 2017).

To achieve a maximal effect on plant–pollinator interactions, the scent-emission machinery has evolved to synchronize with both flower readiness for pollination and the lifestyle of the pollinator (Hoballah et al., 2007). Although some plants present a steady, unchanged emission of volatiles throughout the day, most species possess either diurnal or nocturnal emission patterns that coincide with the activity of their corresponding pollinators (Yon et al., 2017). *Petunia* x *hybrida*, one of the most popular model plants in floral scent research, and its ancestor *Petunia axillaris*, are both pollinated by nocturnal hawkmoths (Ando et al., 2001; Klahre et al., 2011; Sheehan et al., 2012; Bombarely et al., 2016). Petunia floral scent emission increases toward dusk and peaks at night (1900–2300 h), and is nearly undetectable at around noon (Spitzer-Rimon et al., 2010). Production of scent molecules is rhythmical, manifested in diel fluctuations of internal pool sizes and scent-related gene expression, which together orchestrate the characteristic emission pattern (Kolosova et al., 2001; Schuurink et al., 2006; Spitzer-Rimon et al., 2010). This rhythmic mechanism is synchronized by a master gene of the circadian clock, *LATE ELONGATED HYPOCOTYL*, which regulates the expression of the MYB transcription factor *ODORANT1*, which in turn controls the flux of Phe into the phenylpropanoid pathway (Fenske et al., 2015).

Volatile phenylpropanoids and other specialized metabolites, e.g., flavonoids, monoterpenes, betalains, alkalkoids, and brassinosteroids, undergo various postproduction modifications, such as, glycosylation, methylation, and acylation. These modifications increase their stability, enable transport, lower their toxicity by blocking reactive groups and enhance their water solubility, thus enabling traffic and/or storage in subcellular compartments (Li et al., 2001; Bowles et al., 2005; Dean et al., 2005). Extensive research has shown, for example, that non-volatile flavonoids such as, anthocyanins and proanthocyanidins are synthesized on the cytoplasmic surface of the endoplasmic reticulum and stored as glycosides in the central vacuole by transporter-mediated intake, vesicle-mediated transport, or microautophagy (Gomez et al., 2011; Pérez-Díaz et al., 2014; Chanoca et al., 2015). Monolignol-derived lignin monomers, another class of phenylpropanoids, are also stored in the vacuoles as glycosides, most likely prior to their transport into the cell wall where they are polymerized to lignin (Liu, 2012; Dima et al., 2015). As opposed to the substantial knowledge gathered on processes leading to sequestration of flavonoids derived from the phenylpropanoid pathway, much less is known about modifications undergone by floral scent volatiles derived from that same pathway.

Glycosylated scent compounds are generally regarded as storage forms or precursors for the emission of aglycone at the appropriate time or stage of plant or organ development (Rambla et al., 2014). In several plant systems, mono-, di-, and tri-saccharide conjugates of phenylpropanoid volatiles with various types of sugars have been isolated from petals, fruits, and leaves (Guo et al., 1994; Moon et al., 1996; Ortiz-Serrano and Gil, 2010; Zhou et al., 2014; Chen et al., 2015; Yilmaztekin et al., 2015). Glycosylation of volatile scent compounds, like that of other specialized metabolites, is catalyzed by members of a subgroup of the plant multigene family 1 of glycosyltransferases (Bowles et al., 2005; De Bruyn et al., 2015; Tiwari et al., 2016; Brazier-Hicks et al., 2017). For example, two tomato (*Solanum lycopersicum*) fruit glycosyltransferases, *NON-SMOKY GLYCOSYLTRANSFERASE1* (*SlNSGT1*) and *UDP-GLYCOSYLTRANSFERASE 5* (*SlUGT5*), which catalyze the conjugation of sugars to volatile phenylpropanoids, have been characterized in detail (Louveau et al., 2011; Tikunov et al., 2013). In several fruits and flowers, the level of the glycosylated fraction of volatiles is higher than that of the aglycone fraction (Oka et al., 1999; Hayashi et al., 2004; Picone et al., 2004; Tikunov et al., 2010; Chen et al., 2015). In tomato fruit, the emission of several phenylpropanoids is dependent on their hydrolysis from glycosylated moieties (Rambla et al., 2014). In addition, the pool sizes of free volatiles are in direct correlation with those of their conjugates during tomato fruit development (Birtić et al., 2009). Levels of phenylethyl alcohol conjugates, as revealed in studies with rose flowers, decrease in parallel to bud development, as opposed to the free-form phenylethyl alcohol which is only present from anthesis onwards (Watanabe et al., 2001). Phenylethyl alcohol-β-D-glucopyranoside is believed to be the primary precursor for aglycone emission after petal unfurling in rose flowers (Watanabe et al., 2001; Hayashi et al., 2004; Sakai et al., 2008).

Numerous studies have detailed the genetic machinery and biochemical reactions driving production of floral scent in petunia (Muhlemann et al., 2014). These include scent-related regulatory and structural genes, enzymatic steps and metabolic fluxes within branches of the phenylpropanoid pathway. However, no data are available on conjugation or sequestration of petunia volatile phenylpropanoids. Here, we show that petunia flowers produce substantial amounts of glycosylated phenylpropanoid volatiles which, during the light hours, can account for more than 90% of all molecules (aglycone + glycosides) accumulated in petals. These glycosylated volatiles are stored in the central vacuoles of petunia petal cells and their accumulation pattern is positively correlated to flower development. Implications of the floral volatile glycosylation mechanism for scent production are discussed.

## Materials and methods

### Plant material

Rooted petunia plantlets (*Petunia* x *hybrida* L. lines P720, W115 [cv. MD], Blue Spark, and Blue Ray) were obtained from Danziger–Dan Flower Farm (Mishmar Hashiva, Israel). Plants were grown in the greenhouse under a 25/20°C day/night temperature regime and a 12/12 h light/dark photoperiod, with light period starting at 0800 h.

### Collection and GC–MS analysis of free and glycosylated scent compounds accumulated in petals and of emitted volatiles (headspace)

For dynamic headspace analysis (Spitzer-Rimon et al., 2012) of different developmental stages, petunia P720 flowers [ranging from 3.5 cm buds to 3 days postanthesis (DPA)] were collected at 1600 h. For analysis of diel floral emissions, petunia P720 flowers were collected at anthesis stage and headspace was initiated at 0300 h with 4-h intervals for 24 h. Volatiles emitted from detached flowers (three flowers per sample) were collected using an adsorbent trap consisting of a glass tube containing 200 mg Porapak Type Q polymer (80/100 mesh; Alltech) held in place with steel mesh plugs. Trapped volatiles were eluted using 1.5 ml hexane, and 2 μg *iso*butylbenzene was added to each sample as an internal standard. A calibration plot with increasing *iso*butylbenzene concentrations was generated to determine the relative amounts of target volatiles within each sample.

To determine the pool sizes of free volatile compounds (aglycones) in the corolla limbs (Spitzer-Rimon et al., 2012), petal tissues (150 mg fresh weight from at least three different flowers) were collected, ground in liquid nitrogen and extracted in 800 μl hexane containing 2 μg *iso*butylbenzene as the internal standard. Following a 2 h incubation with shaking at 150 rpm, extracts were centrifuged (10,000 g for 10 min) and the supernatant was further centrifuged and evaporated prior to chromatography. Glycosylated scent compounds were extracted by grinding corolla limb tissue (150 mg fresh weight from at least three different flowers) in liquid nitrogen, suspending in 1.2 ml 80% MeOH:double-distilled water (DDW) (v/v) and sonicating for 20 min. Samples were centrifuged (16,000 g, 10 min) and the supernatant was vacuum-dried and then resuspended in 0.9 ml 0.2 M citrate–phosphate buffer, pH 5.4. Viscozyme (150 μl; Sigma Aldrich, ca. 1.2 g/ml) was added to hydrolyze and release the sugar-bound aroma compounds. Following overnight incubation at 37°C with the enzyme, samples were extracted in 800 μl hexane containing 2 μg *iso*butylbenzene as the internal standard, and prepared for GC–MS analysis as described above for aglycone determination. To avoid possible contamination of the preparation with aglycones prior to hydrolysis, we calibrated the protocol for glycoside extractions by extending the evaporation of the samples while heating to promote emission of volatiles. Extraction of samples with an organic solvent after this process, prior to enzymatic hydrolysis of the conjugates, showed no detectable levels of volatiles. For the assessment of diel accumulation of scent compounds (internal pools of aglycones and glycosides), flowers of petunia line P720 were collected between anthesis at 1600 h and 1 DPA at 2400 h, at 8-h intervals for the first day and every 4 h thereafter.

GC–MS analysis (1 μl sample) was performed using a device composed of a Pal autosampler (CTC Analytic), a TRACE GC 2000 equipped with an Rtx-5SIL mass spectrometer fused-silica capillary column (Restek; i.d. 0.25 μm, 30 m × 0.25 mm) and a TRACE DSQ quadruple mass spectrometer (ThermoFinnigan). Helium was used as the carrier gas at a flow rate of 1 ml/min. The injector temperature was set to 220°C (splitless mode) and the interface to 240°C, and the ion source was adjusted to 200°C. The analysis was performed under the following temperature program: 2 min of isothermal heating at 40°C followed by a 7°C/min oven temperature ramp to 250°C then 2 min of isothermal heating. The system was equilibrated for 1 min at 70°C before injection of the next sample. Mass spectra were recorded at 3.15 scan/s with a scanning range of 40–450 mass-to-charge ratio and electron energy of 70 eV. Compounds were tentatively identified (>95% match) based on NIST/EPA/NIH Mass Spectral Library data version NIST 05 (software version 2.0d) using the XCALIBUR v1.3 (ThermoFinnigan). Further identification of major compounds was based on comparison of mass spectra and retention times with those of authentic standards (Sigma-Aldrich) analyzed under similar conditions.

### ^2^H_5_-phe-feeding experiments with detached petunia flowers

Petunia flowers (line P720) were collected 1 DPA at 1500 h. Detached corollas were excised 1.5 cm below the limbs and placed in a 150-μl solution containing 30 mM ^2^H_5_-Phe stable isotope (98% purity, Cambridge Isotope Laboratories). After 20-min incubation, corollas were washed twice with DDW and then transferred to water for the rest of the experiment. Limb samples (150 mg) were collected at different time points (0.5, 2, 4, 6, and 9 h after transfer to water) to determine the internal pool of glycosylated scent compounds (labeled and non-labeled). Detection of conjugated compounds derived from labeled and non-labeled ^2^H_5_-Phe was performed as described by Boatright et al. (2004). Authentic standards were all purchased from Sigma Aldrich.

### Transient overexpression of *AnBGL1* in petunia flowers

Transient overexpression of pBINPLUS carrying CaMV 35S-driven *AnBGL1* coding for *Aspergillus niger* β-glucosidase 1, with or without lytic vacuole-targeting determinants (*AnBGL1vac or AnBGL1cyt*, respectively) cloned at the N-terminus (Wei et al., 2004), was performed in petunia line P720 flowers as described previously (Spitzer-Rimon et al., 2012). Plasmids harboring the BGL1 constructs were sequenced to confirm their validity for further use. Petunia buds 1 day before anthesis were subjected to vacuum infiltration with a mix of *Agrobacterium tumefaciens* strain AGLO carrying pRCS2-35S:*GFP* and bacteria carrying pBINPLUS-35S:*AnBGL1vac* or bacteria carrying pBINPLUS-35S:*AnBGL1cyt* in a 1:1 (v/v) mixture. As a control, buds were inoculated with AGLO carrying only pRCS2-35S:*GFP*. Three days postinfiltration (at 1500 h), open flowers were examined using a fluorescence binocular microscope (FLOUIII, Leica Microsystems) under white and UV light with GFP filters. GFP-positive tissues were excised and glycosylated scent compounds were extracted and analyzed by GC–MS. For β-glucosidase activity assay, tissues (300 mg) were ground in liquid nitrogen and extracted in 1.2 ml 50 mM phosphate buffer pH 4.3, containing 10 mM EDTA and 4 mM dithiothreitol. Total protein level was measured using the Bradford method (Bradford, 1976). Enzyme activity was quantified as in Wei et al. (2004) using pNPG (Sigma Aldrich) as the substrate. Activity was calculated as the amount (in mol) of liberated p-nitrophenyl (pNP) per mg protein per 1 min of reaction time.

### Isolation of protoplasts and intact vacuoles from petunia petals

Protoplasts were isolated from petunia petals (line Blue Ray) as described by Faraco et al. (2014). Protoplast viability was assayed by incubating cells with 50 μg/ml fluorescein diacetate solution (Sigma Aldrich) in a 1:1 mixture (v/v) for 5 min prior to observation under the microscope. Imaging of fluorescent cells was performed by fully motorized epifluorescence inverted microscope (Olympus-IX8 Cell-R) with a 12-bit Orca-AG CCD camera using a GFP filter. Intact vacuoles were isolated from protoplasts as described previously (Robert et al., 2007) with some modifications. Briefly, protoplasts were osmotically and thermally lysed, and the vacuole fraction was separated from the lysate using a Ficoll step gradient in 15-ml tubes. A typical gradient consisted of 5.5 ml lysed protoplasts in 10% (w/v) Ficoll overlain by 4 ml of 5% Ficoll solution and 1.5 ml vacuole buffer (containing 0.25 M mannitol, 5 mM sodium phosphate pH 7.5, and 2 mM EDTA pH 8) layered over that. The tubes were centrifuged for 45 min at 1,500 g. Vacuoles were located between the 0 and 5% Ficoll layers. Vacuole purity was evaluated using light microscopy and western blot analysis (see details below). Anthocyanin content of protoplasts and vacuoles was measured as described by Spitzer-Rimon et al. (2010). Extraction of glycosides from protoplasts and vacuoles and their quantitation by GC–MS were performed as above. To extract proteins for western blot analysis, aliquots of protoplasts and vacuoles were first precipitated in 10% (w/v) trichloroacetic acid, washed with 100% acetone and resuspended in extraction buffer (0.1 M Tris–HCl pH 8, 150 mM dithiothreitol, 2% v/v SDS, 2 mM phenylmethylsulfonyl fluoride, and 5% v/v β-mercaptoethanol). Extracts were further treated as in Vishnevetsky et al. (1999). Briefly, samples were centrifuged and the supernatants were mixed with equal volumes of Tris-saturated phenol (pH 8). After centrifugation, the phenolic phases were washed with the extraction buffer, and 5 volumes of MeOH–ammonium sulfate (0.1 M) were added. Samples were washed three more times with MeOH–ammonium sulfate (0.1 M) and then with 80% acetone. The pellets were resuspended in NuPAGE LDS Sample Buffer (Thermo Fisher Scientific), and the tubes were incubated for 5 min at 55°C prior to electrophoresis. The samples were analyzed on NuPAGE 4–12% Bis–Tris gels (Thermo Fisher Scientific) with the Precision Plus Protein™ All Blue Standards marker (Bio-Rad Laboratories). The volumes of loaded samples from protoplasts and vacuoles were normalized so that they reflected an identical amount of cells/protoplasts. Samples were transferred to iBlot nitrocellulose membranes for western blot analyses and following incubation with skimmed milk-based blocking solution, membranes were reacted overnight at 18°C with the primary antibodies: epsilon subunit of tonoplast V-ATPase and cFBPase (Agrisera, diluted 1:200) (Dima et al., 2015; de Michele et al., 2016), PAL, diluted 1:1,000 (Howles et al., 1996), and LHCII, diluted 1:500 (Vainstein and Sharon, 1993). Following three 10-min washes in Tween–Tris buffered saline, the membranes were incubated for 1 h at room temperature in blocking buffer containing a 1:10,000 dilution of secondary goat anti-rabbit antibody coupled to horseradish peroxidase. After three washes, the membranes were incubated for 1 min with SuperSignal West Pico Chemiluminescent Substrate (Thermo Fisher Scientific) and developed by autoradiography using Image Quant Las 500 (GE Healthcare Bio-Sciences AB).

### Statistical analyses

Significance of differences between treatments were calculated by Student's *t*-test, ^*^P ≤ 0.05, using the SigmaPlot software v13.0 (SYSTAT).

## Results

### Glycosylated scent compounds in cultivars of *Petunia* x *Hybrida*

**Figure 1:**
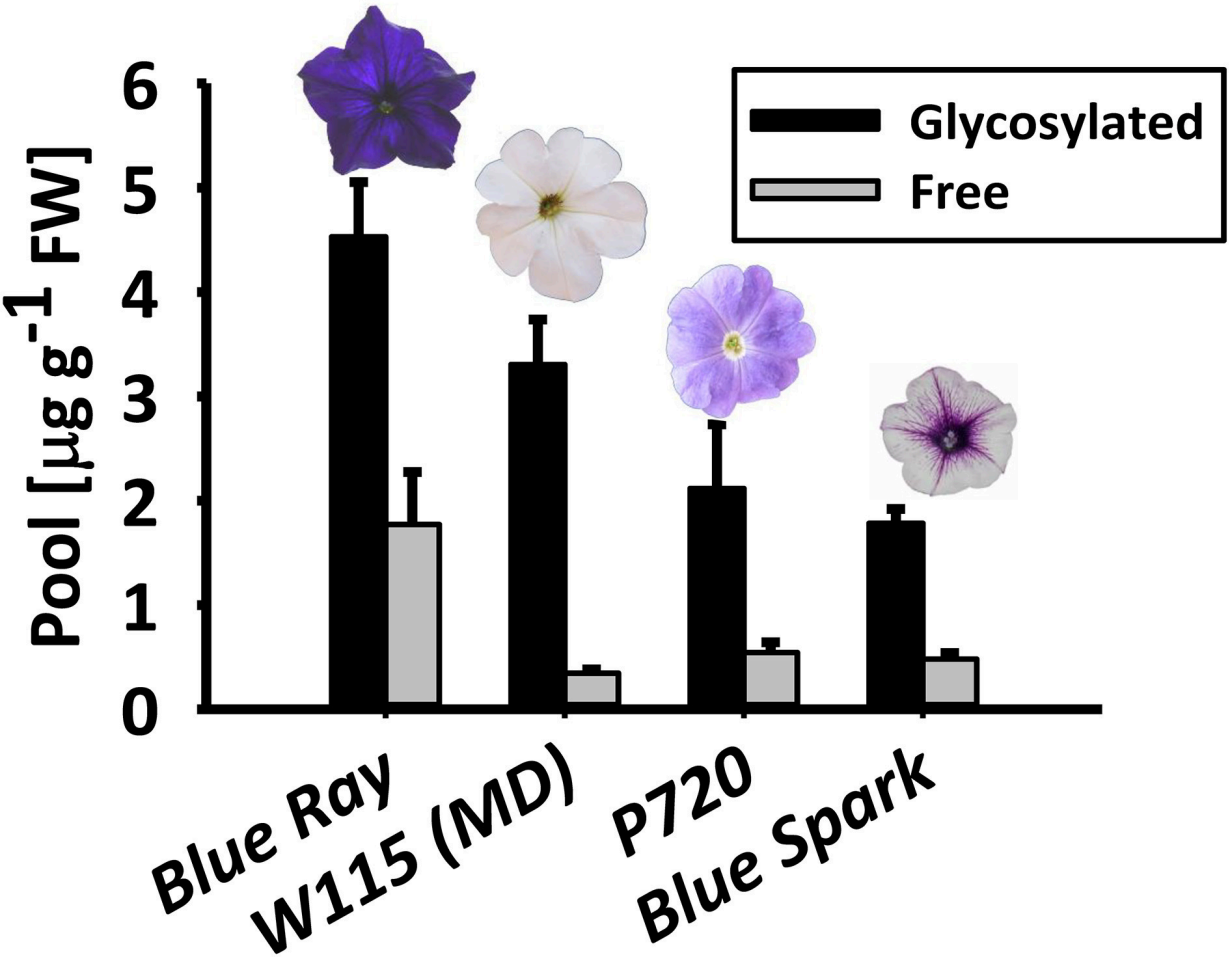
*Petunia* × *hybrida* cultivars accumulate glycosylated phenylpropanoid scent compounds. Glycosylated scent compounds and aglycones from corollas of Blue Ray, W115 (MD), P720, and Blue Spark petunias at anthesis were extracted at 1600 h, and their levels were measured by GC–MS. Columns represent mean values of four to seven independent experiments. SEs are indicated by vertical lines. FW, fresh weight.

To ascertain the prevalence of glycosylated phenylpropanoid volatiles in petunia flowers, their levels were measured using gas chromatography–mass spectrometry (GC–MS) in four *Petunia* x *hybrida* cultivars with different genetic backgrounds: Blue Ray, W115 [Mitchell Diploid (MD)], P720 and Blue Spark. These cultivars are all fragrant (Figure S1), but have different flower shapes, colors, and sizes. Glycosylated and free (non-glycosylated, aglycone) scent compounds were extracted at 1600 h—the onset of scent production—from flowers at anthesis. In all cultivars, the glycosylated fraction of the volatiles, i.e., those with glycosylation-compatible hydroxyl groups (benzyl alcohol, phenylethyl alcohol, eugenol, isoeugenol, and vanillin) was detected as the predominant one, ranging from ~72 to 90% of the combined pool of accumulated (aglycone + glycoside) compounds (Figure 1).

### Accumulation of glycosylated scent compounds during flower development

**Figure 2:**
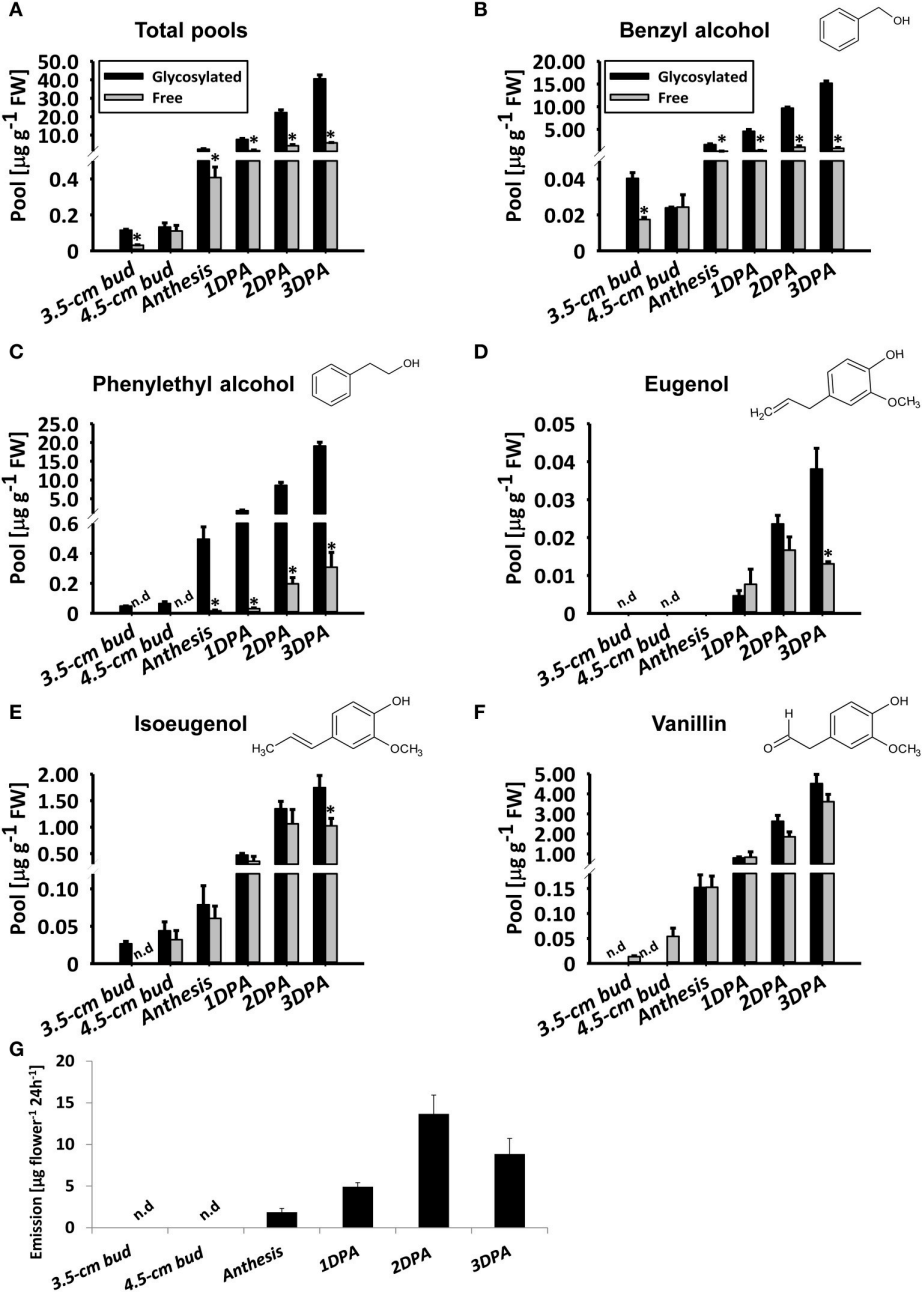
Levels of petunia phenylpropanoid glycosylated scent compounds increase in parallel to flower development. Glycosylated scent compounds and aglycones (Free) from corollas of petunia line P720 were extracted at 1800 h and their levels were measured by GC–MS. Developmental stages of corollas: 3.5 cm buds, 4.5 cm buds, anthesis, and 1, 2, and 3 DPA. **(A–F)** Levels of glycosides and aglycones are presented for **(A)** the sum of the set of scent compounds with glycosylation-compatible hydroxyl groups, consisting of **(B)** benzyl alcohol, **(C)** phenylethyl alcohol, **(D)** eugenol, **(E)** isoeugenol, and **(F)** vanillin. Columns represent the mean values of three to four independent experiments. SEs are indicated by vertical lines. Significance of differences between levels of glycosylated scent compounds and their corresponding aglycones were calculated by Student's *t*-test, ^*^P ≤ 0.05. FW, fresh weight. **(G)** Dynamic headspace analyses of total emitted volatiles followed by GC–MS were performed for 24 h (starting at 1600 h) on P720 petunia corollas at different developmental stages. Columns represent the mean values of three to five independent experiments. SEs are indicated by vertical lines.

To detail the accumulation of glycosylated phenylpropanoid scent compounds, we measured their levels, as well as those of aglycones (accumulated and emitted) in line P720 flowers at different stages of development, at 1800 h. This line was chosen for further examination due to its prolific flowering and in-depth-characterized patterns of diel/developmental scent production (Spitzer-Rimon et al., 2012; Cna'ani et al., 2015; Ravid et al., 2017). As expected (Figure 2A), the level of aglycones increased in parallel to flower development from young bud (3.5 cm) to mature flower. Levels of volatiles emitted from petunia flowers increased similarly until 2DPAand decreased thereafter, as previously reported (Figure 2G and Figure S2; Spitzer-Rimon et al., 2010). Free phenylethyl alcohol, eugenol, and isoeugenol volatiles were below detection levels in young buds (Figures 2C–E). Similar to the aglycones' accumulation and emission, levels of glycosylated scent compounds increased in parallel to flower development (Figure 2A). The total level of glycosides increased up to 3 DPA, reaching 352-fold that in young buds (3.5 cm stage) and 17-fold that at anthesis. Overall, the fraction of glycosides out of the combined pool was 87.5% in flowers 3 DPA. At this stage, with the exception of vanillin (Figure 2F), the proportion of each individual glycoside of a scent compound out of its combined pool (glycosides + aglycones) was highest (Figures 2B–E). Among the individual glycosides, phenylethyl alcohol glycosides showed the most prominent increase, with a 397-fold change from 3.5 cm buds and a 38.5-fold change from anthesis to 3 DPA (Figure 2C). In young buds, glycosylated phenylethyl alcohol and isoeugenol were detected, while the aglycone fraction was not evident (Figures 2C,E). The opposite trend was apparent with vanillin: only aglycones and no glycosides were detected at early stages of flower development (Figure 2F). No emission of volatiles could be detected in young flower buds (Figure S2).

### Diel accumulation pattern of glycosylated phenylpropanoid volatiles

**Figure 3:**
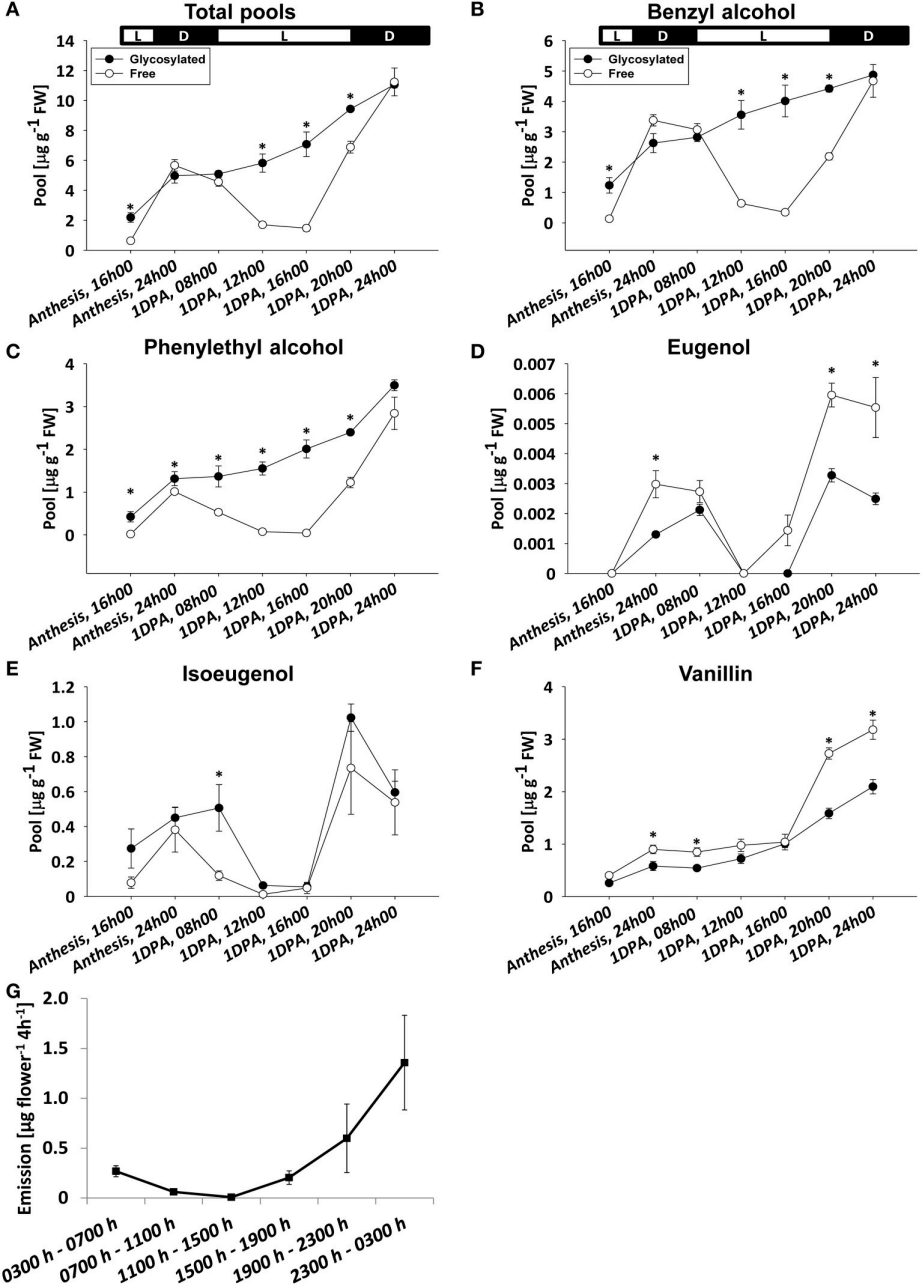
Diel accumulation pattern of glycosylated scent compounds and their corresponding aglycones. Glycosylated scent compounds and aglycones (Free) from petunia corollas were extracted at set intervals during the first 32 h after flower opening (every 8 h during anthesis and every 4 h for 1 DPA) and their levels were analyzed by GC–MS. **(A–F)** Levels of glycosides and aglycones are presented for **(A)** the sum of the set of scent compounds with glycosylation-compatible hydroxyl groups, consisting of **(B)** benzyl alcohol, **(C)** phenylethyl alcohol, **(D)** eugenol, **(E)** isoeugenol, and **(F)** vanillin. Each time point represents the mean values of three to four independent experiments. SEs are indicated by vertical lines. Significance of differences between levels of glycosylated scent compounds and their corresponding aglycones were calculated by Student's *t*-test, ^*^P ≤ 0.05. FW, fresh weight. Black parts of horizontal bars indicate dark hours, white parts represent light periods. **(G)** Dynamic headspace analyses of total emitted volatiles followed by GC–MS were performed for 24 h at 4-h intervals (starting at 0300 h, anthesis) on P720 petunia corollas. Each time point represents the mean values of three to four independent experiments. SEs are indicated by vertical lines.

To characterize the diel accumulation pattern of glycosylated scent compounds and to assess its relevance to the rhythmic nature of scent production, volatile compounds accumulated in and emitted from flowers of petunia line P720 were sampled between anthesis and 1 DPA. Glycosides and corresponding aglycones were sampled in 8-h intervals at anthesis and 4-h during the following day. Headspace analysis was conducted with 4-h intervals for 24 h. GC–MS analyses of the aglycone fraction of phenylpropanoid scent compounds in the pool and headspace (Figures 3A,G, and Figure S3) revealed a typical oscillating pattern during the day, with maximum amplitude levels at around midnight and low points at midday (Oyama-Okubo et al., 2005; Fenske et al., 2015). The peak level of aglycones in the pool at 2400 h 1 DPA was 2-fold higher than at anthesis. As with scent emission (Figure 2G and Figure S3), between the end of the dark period and 1200 h there was a decrease, whereas from 1600 h onward there was a sharp increase in aglycones' internal pool levels. GC–MS analyses of the collective pool of glycosylated scent compounds revealed a different accumulation pattern: following anthesis, total pool size continued to increase from the morning hours throughout the time frame of the experiment. Highest levels of glycosides each day were detected during the dark period, with a 2.2-fold increase 1 DPA vs. anthesis at 2400 h (Figure 3A). The ratio between total glycosylated and free forms of volatiles was highest during the day (1200 to 1600 h), when accumulated aglycones and emission levels were lowest. At those time points, the glycosylated forms accounted for as much as ~70 and 80% of the combined pool, respectively. The lowest ratios were measured at midnight, when the level of free volatiles accumulated in the petals, and of those emitted, peaked. Analyses of individual floral scent compounds revealed that the diel accumulation pattern of total glycosides is largely determined by the levels of benzyl alcohol, phenylethyl alcohol, and vanillin, which together constituted up to 90% of all glycosides within the internal pool of petunia flowers (Figures 3B,C,F). Glycosides of eugenol and isoeugenol, comprising 10–25% of total glycosides (depending on the time of day), accumulated in a pattern that was somewhat similar to their corresponding aglycones, i.e., lower levels around midday and higher levels at night (Figures 3D,E).

### Kinetics of volatile glycoside metabolism in petunia flowers

**Figure 4:**
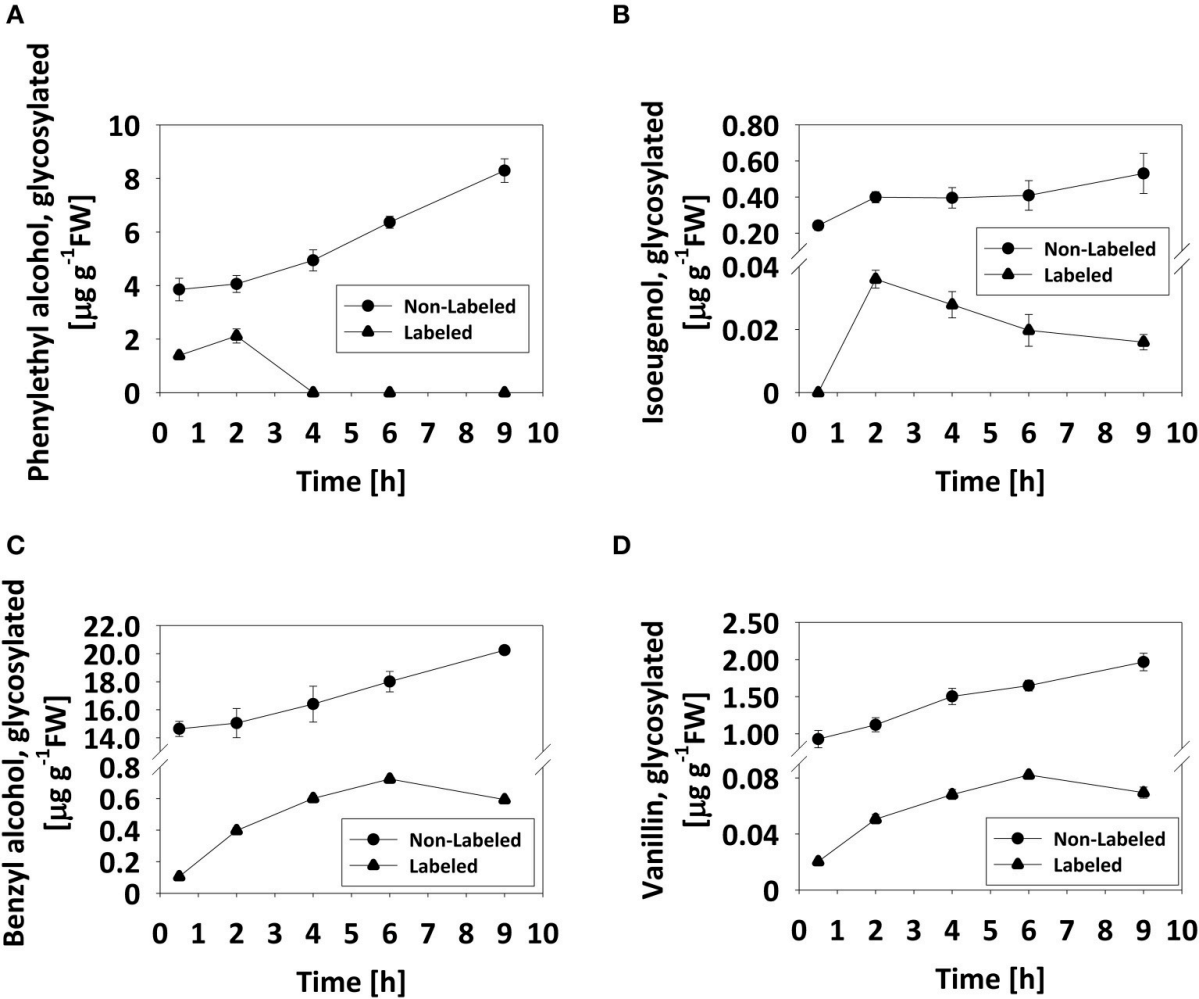
Pool of glycosylated scent compounds is dynamic. Detached petunia corollas (1 DPA, 1500 h) were briefly (20 min) fed with ^2^H_5_-Phe (0) and then transferred to water. Glycosylated scent compounds were extracted 0.5, 2, 4, 6, and 9 h after transfer to water and the levels of labeled and non-labeled glycosides were analyzed by GC–MS. Each time point represents the mean value of four to five independent experiments. SEs are indicated by vertical lines. **(A–D)** Kinetics of labeled (•) and non-labeled (▴) glycosides are presented for **(A)** phenylethyl alcohol, **(B)** isoeugenol, **(C)** benzyl alcohol, and **(D)** vanillin.

Based on the observation that the levels of glycosylated benzyl alcohol, phenylethyl alcohol, and vanillin do not decrease during flower development or on a diel basis (Figures 2, 3), we tested whether the glycosylated pools are dynamic, i.e., that they are not simply sequestered for storage but rather also catabolized during scent production. To monitor changes in glycosides, labeling experiments using deuterium ring-labeled Phe (^2^H_5_-Phe) were performed. This stable isotope has been used to elucidate the pathways leading to benzenoid formation in petunia flowers (Boatright et al., 2004). Flowers of petunia line P720, collected 1 DPA at 1500 h, were fed for 20 min with a solution containing ^2^H_5_-Phe, and changes in the levels of labeled glycosides were analyzed by GC–MS over the following 9 h. The time frame of the experiment was chosen to reflect both the onset and peak in scent production. Preliminary experiments had shown that a short feeding time (20 min) is sufficient to label almost 99% of the pool of phenylacetaldehyde, the first volatile metabolite directly derived from Phe (Figure S4; Farhi et al., 2010). The detection of labeled phenyethyl alcohol, benzyl alcohol, vanillin, and isoeugenol glycosides, and the discrimination between labeled and non-labeled compounds are presented in Figures S5A–D. Labeled eugenol glycosides were not detected in our experimental setup, given that the level of eugenol glycoside is <1% of those of other detectable glycosides and its production necessitates many additional enzymatic steps as compared to, for example, phenyethyl alcohol. Levels of non-labeled glycosides of all scent compounds increased, as expected, during the course of the experiment (Figure 4). Levels of labeled phenyethyl alcohol and isoeugenol glycosides peaked 2 h after feeding with ^2^H_5_-Phe; the former then sharply decreased to undetectable levels, while the latter decreased gradually (Figures 4A,B). Levels of labeled benzyl alcohol and vanillin glycosides also increased after feeding and then decreased 6 h after labeling (Figures 4C,D).

### Transient expression of a β-glucosidase targeted to the vacuole

**Figure 5:**
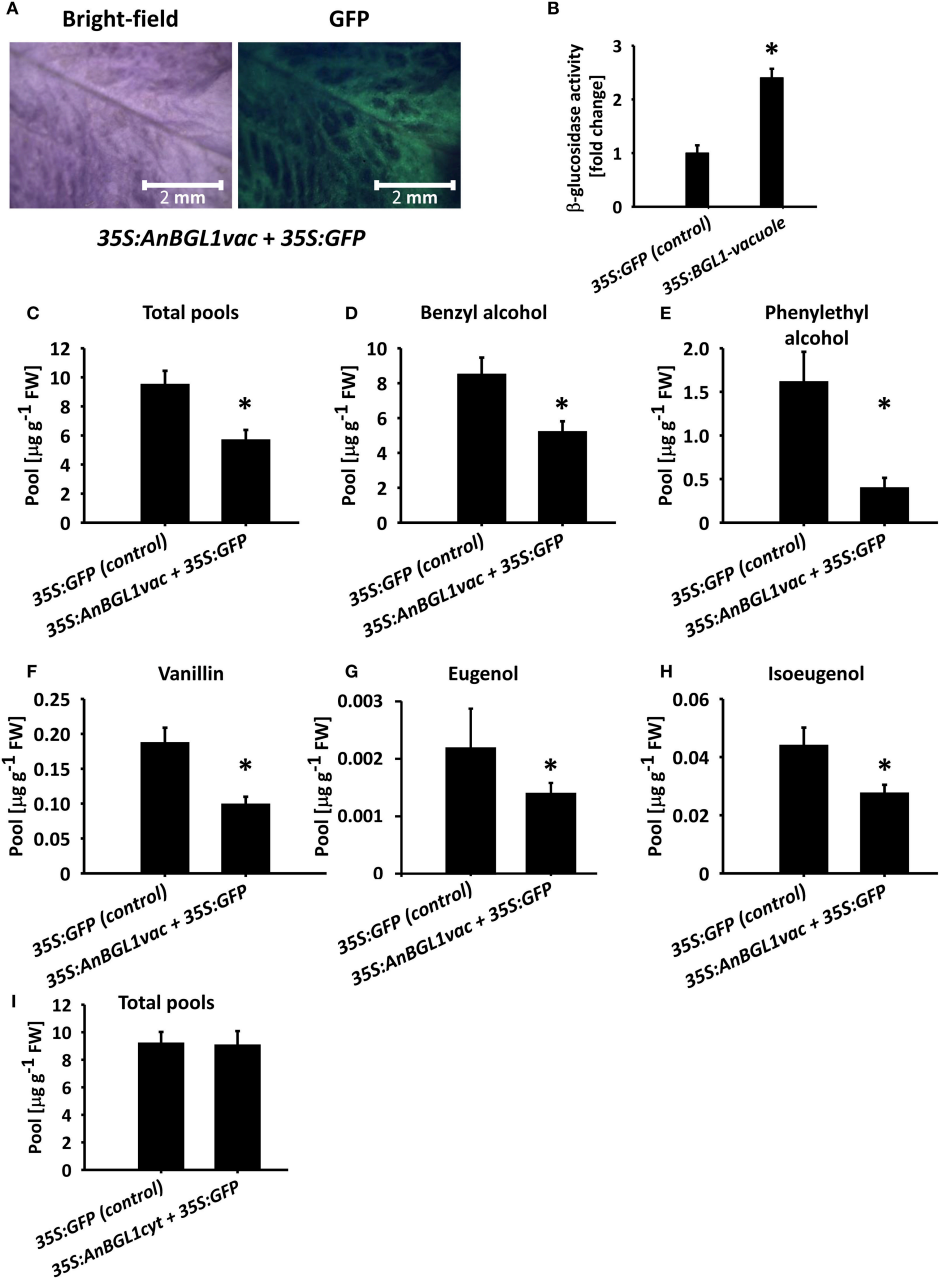
Targeting of *aspergillus niger* β*-glucosidase 1* to the vacuole (*AnBGL1vac*) reduces the levels of glycosylated phenylpropanoid scent compounds. Petunia buds (4 cm, 1 day before anthesis) were agro-infiltrated with 35S:*AnBGL1vac* + 35S:*GFP* or pRCS2-35S:*GFP* alone as a control. Three days postinoculation, glycosylated scent compounds and aglycones were extracted from corollas (1500 h) and analyzed by GC–MS. **(A)** Bright-field and fluorescent (GFP filter) images of pBINPLUS-35S:*AnBGL1vac* + pRCS2-35S:*GFP*-infiltrated petals were taken with a fluorescence binocular microscope under white and UV light with GFP filters at x5 zoom. **(B)** β-glucosidase activity assay in protein extracts from petals infiltrated with pBINPLUS-35S:*AnBGL1vac* + pRCS2-35S:*GFP* and pRCS2-35S:*GFP* (control). Values are normalized to the amount of total protein in the samples. Columns represent the mean values of three independent experiments. SEs are indicated by vertical lines. Significance of differences between treatments was calculated by Student's *t*-test, ^*^P ≤ 0.05. **(C–H)** Levels of glycosides in petals infiltrated with pBINPLUS-35S:*AnBGL1vac* + pRCS2-35S:*GFP* and pRCS2-35S:*GFP* (control) are presented for **(C)** the sum of the set of scent compounds with glycosylation-compatible hydroxyl groups, consisting of **(D)** benzyl alcohol, **(E)** phenylethyl alcohol, **(F)** vanillin, **(G)** eugenol, and **(H)** isoeugenol. **(I)** Levels of the sum of glycosides and aglycones in corollas inoculated with cytosol-targeted β-glucosidase (35S:*AnBGL1cyt*). Corollas were infiltrated with pBINPLUS-35S:*AnBGL1cyt* + pRCS2-35S:*GFP* or pRCS2-35S:*GFP* (control) followed by GC–MS analyses. Columns represent the mean values of four to six independent experiments. SEs are indicated by vertical lines. Significance of differences between treatments was calculated by Student's *t*-test, ^*^P ≤ 0.05.

Flavonoids, such as, anthocyanins, proanthocyanidins, and monolignol-derived lignin monomers, as well as coumarins, hydroxycinnamates, and salicylic acid accumulate as glycosides in the central vacuole (Dean et al., 2005; Chanoca et al., 2015; Dima et al., 2015; Le Roy et al., 2016). To evaluate the vacuole as the subcellular accumulation site of glycosylated volatile phenylpropanoids, we took an indirect approach because methods for determining intracellular localization of small molecules are not yet widely available (Hicks and Raikhel, 2009). We transiently expressed vacuole- or cytosol-targeted versions of β-glucosidase from *A. niger* (pBINPLUS-35S:*AnBGL1vac;* Wei et al., 2004) in 4 cm petunia buds to test whether it would lead to a reduction in glycosylated scent compounds accumulating in petals. Green fluorescent protein (GFP) was expressed with or without β-glucosidase to facilitate the identification of inoculated tissues (Figure 5A). Transient expression of *AnBGL1vac* led to a ca. 2.4-fold increase in β-glucosidase activity compared to control flowers expressing GFP alone, as measured by *p*-nitrophenyl glucopyranoside (pNPG) substrate assay of petal extracts 3 days after inoculation (Figure 5B). GC–MS analyses of the 35S:*AnBGL1vac*-expressing flowers revealed the accumulation of significantly less glycosylated scent compounds compared to control flowers (Figure 5C) with a concomitant increase in levels of the corresponding aglycones (Figure S6). It should be noted that isoeugenol and eugenol aglycones were not detected in either treatment, probably due to their low levels at this time of day. This trend was evident with each individual compound, namely benzyl alcohol, phenyethyl alcohol, vanillin, eugenol, and isoeugenol (Figures 5D–H). Inoculation of petunia flowers with 35S:*AnBGL1* targeted to the cytosol (pBINPLUS-35S:*AnBGL1cyt*) did not affect the levels of glycosylated scent compounds (Figure 5I).

### Glycosides of volatile phenylpropanoids in intact vacuoles isolated from petunia petal protoplasts

**Figure 6:**
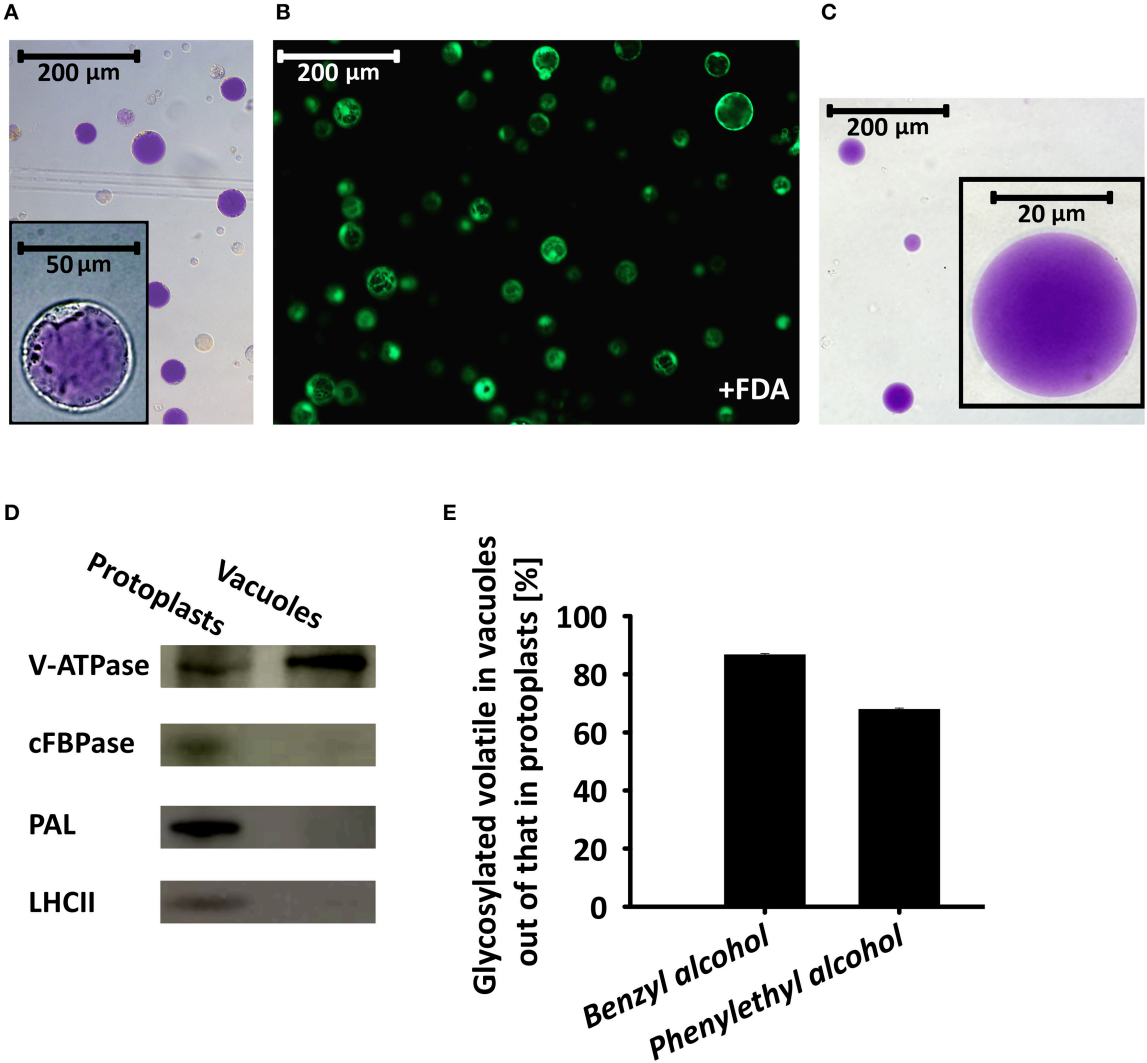
Glycosylated scent compounds are stored in the central vacuoles of petunia petal cells. **(A)** Light microscopy of protoplasts isolated from petals at anthesis. Inset, zooming in on one protoplast. **(B)** Fluorescence microscopy (GFP filter) of fluorescein diacetate (FDA)-stained protoplasts (viability assay). **(C)** Light microscopy inspection of intact vacuoles isolated from protoplasts following chemical and thermal lysis and 0–10% Ficoll step gradient. All images were taken with a fully motorized epifluorescence inverted microscope with a 12-bit CCD camera using a GFP filter with Xcellence RT program v1.2. **(D)** Western blot analysis of proteins extracted from protoplasts and vacuoles and separated by SDS–PAGE, using antibodies against the following cellular protein markers: epsilon subunit of tonoplast V-type H^+^ATPase (V-ATPase, tonoplast), cytosolic fructose-1,6 bisphosphatase (cFBPase, cytosol), phenylalanine ammonia lyase (PAL, endomembrane/cytosol) and major light-harvesting chlorophyll a/b protein (LHCII, plastid). **(E)** GC–MS analyses of benzyl alcohol and phenylethyl alcohol glycosides extracted from protoplasts and vacuoles. Glycoside levels were normalized to anthocyanin content and their levels in protoplasts were set at 100%. This experiment was conducted twice.

To further corroborate the results implicating the vacuole as the storage site for glycosides of phenylpropanoid volatiles, we isolated intact vacuoles for GC–MS analysis. About 90% of the protoplasts used for the isolation of vacuoles were found to be viable, as evaluated by fluorescein diacetate staining (Figure 6B). Intact pure vacuoles (based on light microscopy observation, Figure 6A, inset and Figure 6C) were isolated from these protoplasts with an efficiency of 10–15% based on protoplast vs. vacuole numbers or their anthocyanin contents. Purity of the vacuole isolation was further confirmed by western blot analysis with antibodies against the epsilon subunit of tonoplast V-type H^+^ATPase (V-ATPase, tonoplast), cytosolic fructose-1,6 bisphosphatase (cFBPase, cytosol), phenylalanine ammonia lyase (PAL, endomembrane/cytosol), and major light-harvesting chlorophyll a/b protein (LHCII, plastid; Vainstein and Sharon, 1993; Howles et al., 1996; Dima et al., 2015; de Michele et al., 2016). V-ATPase signal was detected in protein extracts of the vacuoles, whereas there was no signal from antibodies against markers of other cellular compartments. Proteins extracted from protoplasts cross-reacted, as expected, with all of the aforementioned antibodies (Figure 6D). GC–MS analysis of the vacuole fraction revealed pools of benzyl alcohol and phenylethyl alcohol glycosides. These were the dominant metabolites in the pool of glycosides of phenylpropanoid floral scent compounds (amounting to ca. 80%, Figure 2), and were the only ones that could also be detected in the protoplast fraction. Most of protoplasts' benzyl alcohol and phenylethyl alcohol glycosides were detected in vacuoles. Normalized to anthocyanin levels, 86.7% of the former and 67.9% of the latter were found in vacuoles (Figure 6E).

## Discussion

Glycosylation is often employed for intracellular trafficking and storage of specialized metabolites within the cell, since it decreases the compound's activity by blocking reactive groups, increases its water solubility, reduces its volatility, and serves as a signal for translocation (Bowles et al., 2005; Gachon et al., 2005; Tiwari et al., 2016; Szeja et al., 2017). Glycoconjugation of scent compounds is highly intriguing because it renders these metabolites non-volatile and they can potentially serve as a readily hydrolyzed pool for the emission of aglycones, adding another layer of complexity to the machinery regulating floral scent production. Petunia is one of the leading model plants for floral scent research, and has been used in numerous studies focusing on the biochemistry and genetics of this trait (Bombarely et al., 2016; Vandenbussche et al., 2016); however, as opposed to other model plant systems in floral scent research (e.g., roses), no data are available on the degree of glycosylation of Phe-derived volatiles in flowers of this plant.

Using the rapid and insightful analytical method of GC–MS on hydrophilic petal extracts that have undergone dehydration, heating, and enzymatic hydrolysis, we revealed that flowers of several petunia cultivars produce and accumulate substantial amounts of glycosylated phenylpropanoid scent compounds, reaching up to 90% of all scent compounds (combined pool of aglycones + glycosides) in the tissue at the onset of scent emission (Figure 1). We preferred this method over liquid chromatography (LC)–MS analyses because targeted profiling requires authentic standards (which are unknown in petunia) and non-targeted metabolomics is time-consuming. Our results indicated that petunia flowers accumulate far more volatiles that undergo glycosylation (namely benzyl alcohol, phenylethyl alcohol, vanillin, isoeugenol, and eugenol) than previously reported. These pools of conjugated scent compounds may potentially be of use in the metabolic enhancement of petunia aroma. The occurrence of glycosides as the major form of pooled volatiles has also been demonstrated in other plants, such as, phenylethyl alcohol glycosides in rose (*Rosa damascena*) flowers (Oka et al., 1999; Hayashi et al., 2004; Picone et al., 2004), eugenol glycosides in tomato (*S. lycopersicum*) fruit (Tikunov et al., 2010), and benzyl alcohol and phenylethyl alcohol glycosides in tea (*Camellia sinensis*) leaves (Zhang et al., 2006).

In rose flowers, levels of phenylethyl alcohol glycosides decrease as the light period progresses, while the level of accumulated aglycones does not change (Oka et al., 1999; Sakai et al., 2008). In *Nicotiana sylvestris, Nicotiana suaveolens* (Loughrin et al., 1992), and *Trifolium repens* (Jakobsen and Christensen, 2002) flowers, on the other hand, levels of glycosylated phenylpropanoid scent compounds do not seem to change markedly during the day. Analyses of the diel accumulation pattern of major glycosylated scent compounds in petunia (benzyl alcohol, phenylethyl alcohol, and vanillin) revealed that their level is essentially the same between midnight and the beginning of the light period. At that point, it starts to increase (in parallel to the reduction in aglycone level) and continues to rise until midnight, when scent emission peaks (Figure 3; Spitzer-Rimon et al., 2010). Furthermore, while the levels of all aglycones oscillate during the day/night, peaking at 2400 h, those of the glycosides do not decrease. Levels of eugenol and isoeugenol in petunia flowers present yet another pattern of diel accumulation. Based on the different accumulation patterns of these glycoside/aglycone scent compounds, we suggest that these water-soluble glycosylated compounds can serve as a means to sequester volatile compounds inside the cell, when scent emission is at its lowest point, in synchronization with pollinator activity.

Numerous compounds, such as, ions, sugars, proteins, amino acids, and specialized metabolites—including phenylpropanoids—are stored in vacuoles (Zhang et al., 2014; Fettke and Fernie, 2015; Lynch et al., 2017). Moreover, phenylpropanoid-derived metabolites such as, anthocyanins, coumarins, salicylic acid, and monolignols are stored as glycosides in the vacuole (Widhalm et al., 2015; Le Roy et al., 2016). Anthocyanidins are glycosylated in the cystosol prior to their transport to the vacuoles via at least three main routes, providing evidence for the importance and complexity of intracellular trafficking of specialized metabolites (Gomez et al., 2011; Francisco et al., 2013; Zhao et al., 2014; Chanoca et al., 2015). The case of monolignol glycosides, the precursors of lignin and lignan/(neo)lignan formation, is even more intriguing, because these compounds are destined for the apoplast. These metabolites undergo combinatorial cross-coupling reactions in the cytosol or in the cell wall where lignin is deposited (Liu, 2012; Dima et al., 2015). Alternatively, monolignols and oligolignols are first glycosylated in the cytoplasm (Dima et al., 2015), sequestered in the vacuole (Miao and Liu, 2010), and then most likely translocated to their final destination in the cell wall (Lin et al., 2016). Similarly, in *Melilotus albus* leaves, coumarin glycosides are stored in the vacuole, while the corresponding dedicated hydrolyzing enzyme is localized to the cell wall (Oba et al., 1981).)Using two independent strategies, we showed that phenylpropanoid scent compounds are also stored as glycosides in the central vacuole of petunia petal cells: overexpression of *AnBGL1* targeted to the vacuole, which resulted in a decrease in the level of said glycosides; isolation of intact vacuoles from protoplasts of petal cells and GC–MS analysis of their contents. The content of anthocyanin, which is stored exclusively in the vacuoles, was used to normalize the level of glycosides between protoplasts and vacuoles. GC–MS analyses revealed that the vacuoles contain 86.7 and 67.9% of the cell's pool of benzyl alcohol and phenylethyl alcohol glycosides, respectively. It may be that due to experimental limitations, we could not recover 100% of the protoplasts' benzyl alcohol and phenylethyl alcohol glycosides in the vacuole; alternatively, this difference in amounts may reflect molecules that are en route to or from the vacuole or those accumulating in alternative intracellular spaces.

We suggest that glycosylated metabolites originating from distinct branches of the phenylpropanoid pathway share a strategy with respect to vacuolar sequestration. We hypothesize that conjugated scent compounds, similar to other classes of Phe-derived metabolites, are retained in the vacuoles not only as a surplus or detoxification mechanism, but also as a station on their route to secretion. Feeding experiments revealed that the pool of glycosylated compounds is metabolized during peak scent emission. The dynamic nature of this pool supports the possibility of it being harnessed for scent production.

## Author contributions

AC and AV conceived the project and research plan and designed the experiments; AC performed most of the experiments, analyzed the data, and wrote the manuscript; RS, JR, JA, OS, and TM provided technical support and data analyses and performed some of the experiments.

### Conflict of interest statement

The authors declare that the research was conducted in the absence of any commercial or financial relationships that could be construed as a potential conflict of interest.

## References

[B1] Adebesin F. Widhalm J. R. Boachon B. Lefèvre F. Pierman B. Lynch J. H.. (2017). Emission of volatile organic compounds from petunia flowers is facilitated by an ABC transporter. Science 356, 1386–1388. 10.1126/science.aan0826 28663500

[B2] Ando T. Nomura M. Tsukahara J. Watanabe H. Kokubun H. Tsukamoto T. (2001). Reproductive isolation in a native population of Petunia sensu Jussieu (Solanaceae). Ann. Bot. 88, 403–413. 10.1006/anbo.2001.1485

[B3] Birtić S. Ginies C. Causse M. Renard C. M. G. C. Page D. (2009). Changes in volatiles and glycosides during fruit maturation of two contrasted tomato (*Solanum lycopersicum*) lines. J. Agric. Food Chem. 57, 591–598. 10.1021/jf8023062 19154163

[B4] Boatright J. Negre F. Chen X. Kish C. M. Wood B. Peel G.. (2004). Understanding *in vivo* benzenoid metabolism in petunia petal tissue. Plant Physiol. 135, 1993–2011. 10.1104/pp.104.045468 15286288 PMC520771

[B5] Bombarely A. Moser M. Amrad A. Bao M. Bapaume L. Barry C. S.. (2016). Insight into the evolution of the Solanaceae from the parental genomes of *Petunia hybrida*. Nat. Plants 2:16074. 10.1038/nplants.2016.74 27255838

[B6] Bowles D. Isayenkova J. Lim E.-K. Poppenberger B. (2005). Glycosyltransferases: managers of small molecules. Curr. Opin. Plant Biol. 8, 254–263. 10.1016/j.pbi.2005.03.007 15860422

[B7] Bradford M. M. (1976). A rapid and sensitive method for the quantitation of microgram quantities of protein utilizing the principle of protein-dye binding. Anal. Biochem. 72, 248–254. 10.1016/0003-2697(76)90527-3 942051

[B8] Brazier-Hicks M. Gershater M. Dixon D. Edwards R. (2017). Substrate specificity and safener inducibility of the plant UDP-glucose-dependent family 1 glycosyltransferase super-family. Plant Biotechnol. J. [Epub ahead of print]. 10.1111/pbi.12775 28640934 PMC5785338

[B9] Chanoca A. Kovinich N. Burkel B. Stecha S. Bohorquez-Restrepo A. Ueda T.. (2015). Anthocyanin vacuolar inclusions form by a microautophagy mechanism. Plant Cell 27, 2545–2559. 10.1105/tpc.15.00589 26342015 PMC4815043

[B10] Chen L. Zhang X. Jin Q. Yang L. Li J. Chen F. (2015). Free and bound volatile chemicals in Mulberry (*Morus atropurpurea* Roxb.). J. Food Sci 80, C975–C982. 10.1111/1750-3841.12840 25817411

[B11] Cna'ani A. Mühlemann J. K. Ravid J. Masci T. Klempien A. Nguyen T. T. H.. (2015). *Petunia* × *hybrida* floral scent production is negatively affected by high-temperature growth conditions. Plant Cell Environ. 38, 1333–1346. 10.1111/pce.12486 25402319

[B12] De Bruyn F. Maertens J. Beauprez J. Soetaert W. De Mey M. (2015). Biotechnological advances in UDP-sugar based glycosylation of small molecules. Biotechnol. Adv. 33, 288–302. 10.1016/j.biotechadv.2015.02.005 25698505

[B13] de Michele R. McFarlane H. E. Parsons H. T. Meents M. J. Lao J. González Fernández-Ni-o S. M.. (2016). Free-flow electrophoresis of plasma membrane vesicles enriched by two-phase partitioning enhances the quality of the proteome from Arabidopsis seedlings. J. Proteome Res. 15, 900–913. 10.1021/acs.jproteome.5b00876 26781341

[B14] Dean J. V. Mohammed L. A. Fitzpatrick T. (2005). The formation, vacuolar localization, and tonoplast transport of salicylic acid glucose conjugates in tobacco cell suspension cultures. Planta 221, 287–296. 10.1007/s00425-004-1430-3 15871031

[B15] Dima O. Morreel K. Vanholme B. Kim H. Ralph J. Boerjan W. (2015). Small glycosylated lignin oligomers are stored in Arabidopsis leaf vacuoles. Plant Cell 27, 695–710. 10.1105/tpc.114.134643 25700483 PMC4558659

[B16] Faraco M. Spelt C. Bliek M. Verweij W. Hoshino A. Espen L.. (2014). Hyperacidification of vacuoles by the combined action of two different P-ATPases in the tonoplast determines flower color. Cell Rep. 6, 32–43. 10.1016/j.celrep.2013.12.009 24388746

[B17] Farhi M. Lavie O. Masci T. Hendel-Rahmanim K. Weiss D. Abeliovich H.. (2010). Identification of rose phenylacetaldehyde synthase by functional complementation in yeast. Plant Mol. Biol. 72, 235–245. 10.1007/s11103-009-9564-0 19882107

[B18] Fenske M. P. Hewett Hazelton K. D. Hempton A. K. Shim J. S. Yamamoto B. M. Riffell J. A.. (2015). Circadian clock gene LATE ELONGATED HYPOCOTYL directly regulates the timing of floral scent emission in Petunia. Proc. Natl. Acad. Sci. U.S.A. 112, 9775–9780. 10.1073/pnas.1422875112 26124104 PMC4534231

[B19] Fettke J. Fernie A. R. (2015). Intracellular and cell-to-apoplast compartmentation of carbohydrate metabolism. Trends Plant Sci. 20, 490–497. 10.1016/j.tplants.2015.04.012 26008154

[B20] Francisco R. M. Regalado A. Ageorges A. Burla B. J. Bassin B. Eisenach C.. (2013). ABCC1, an ATP binding cassette protein from grape Berry, transports anthocyanidin 3-O-glucosides. Plant Cell 25, 1840–1854. 10.1105/tpc.112.102152 23723325 PMC3694709

[B21] Gachon C. M. M. Langlois-Meurinne M. Saindrenan P. (2005). Plant secondary metabolism glycosyltransferases: the emerging functional analysis. Trends Plant Sci. 10, 542–549. 10.1016/j.tplants.2005.09.007 16214386

[B22] Gaffal K. P. (2012). How common is the ability of extrafloral nectaries to produce nectar droplets, to secrete nectar during the night and to store starch? Plant Biol. 14, 691–695. 10.1111/j.1438-8677.2012.00616.x 22672217

[B23] Gomez C. Conejero G. Torregrosa L. Cheynier V. Terrier N. Ageorges A. (2011). *In vivo* grapevine anthocyanin transport involves vesicle-mediated trafficking and the contribution of anthoMATE transporters and GST. Plant J. 67, 960–970. 10.1111/j.1365-313X.2011.04648.x 21605207

[B24] Guo W. Hosoi R. Sakata K. Watanabe N. Yagi A. Ina K.. (1994). (S)-Linalyl, 2-phenylethyl, and benzyl disaccharide glycosides isolated as aroma precursors from oolong tea leaves. Biosci. Biotechnol. Biochem. 58, 1532–1534. 10.1271/bbb.58.1532 7522061

[B25] Hayashi S. Yagi K. Ishikawa T. Kawasaki M. Asai T. Picone J. (2004). Emission of 2-phenylethanol from its β-d-glucopyranoside and the biogenesis of these compounds from [^2^H8] L-phenylalanine in rose flowers. Tetrahedron 60, 7005–7013. 10.1016/j.tet.2003.10.130

[B26] Hicks G. R. Raikhel N. V. (2009). Opportunities and challenges in plant chemical biology. Nat. Chem. Biol. 5, 268–272. 10.1038/nchembio0509-268 19377447

[B27] Hoballah M. E. Gübitz T. Stuurman J. Broger L. Barone M. Mandel T.. (2007). Single gene-mediated shift in pollinator attraction in Petunia. Plant Cell 19, 779–790. 10.1105/tpc.106.048694 17337627 PMC1867374

[B28] Howles P. A. Sewalt V. J. H. Paiva N. L. Elkind Y. Bate N. J. Lamb C.. (1996). Overexpression of L-phenylalanine ammonia-lyase in transgenic tobacco plants reveals control points for flux into phenylpropanoid biosynthesis. Plant Physiol. 112, 1617–1624. 10.1104/pp.112.4.1617 12226468 PMC158095

[B29] Jakobsen H. B. Christensen L. P. (2002). Diurnal changes in the concentrations of 2-phenylethyl β-D-glucopyranoside and the corresponding volatile aglycone in the tissue and headspace of *Trifolium repens* L. florets. Plant Cell Environ. 25, 773–781. 10.1046/j.1365-3040.2002.00855.x

[B30] Kaminaga Y. Schnepp J. Peel G. Kish C. M. Ben-Nissan G. Weiss D.. (2006). Plant phenylacetaldehyde synthase is a bifunctional homotetrameric enzyme that catalyzes phenylalanine decarboxylation and oxidation. J. Biol. Chem. 281, 23357–23366. 10.1074/jbc.M602708200 16766535

[B31] Klahre U. Gurba A. Hermann K. Saxenhofer M. Bossolini E. Guerin P. M.. (2011). Pollinator choice in petunia depends on two major genetic loci for floral scent production. Curr. Biol. 21, 730–739. 10.1016/j.cub.2011.03.059 21497087

[B32] Klempien A. Kaminaga Y. Qualley A. Nagegowda D. A. Widhalm J. R. Orlova I.. (2012). Contribution of CoA ligases to benzenoid biosynthesis in petunia flowers. Plant Cell 24, 2015–2030. 10.1105/tpc.112.097519 22649270 PMC3442584

[B33] Kolosova N. Gorenstein N. Kish C. M. Dudareva N. (2001). Regulation of circadian methyl benzoate emission in diurnally and nocturnally emitting plants. Plant Cell 13, 2333–2347. 10.1105/tpc.13.10.2333 11595805 PMC139162

[B34] Le Roy J. Huss B. Creach A. Hawkins S. Neutelings G. (2016). Glycosylation is a major regulator of phenylpropanoid availability and biological activity in plants. Front. Plant Sci. 7:735. 10.3389/fpls.2016.00735. 27303427 PMC4880792

[B35] Li Y. Baldauf S. Lim E.-K. Bowles D. J. (2001). Phylogenetic analysis of the UDP-glycosyltransferase multigene family of *Arabidopsis thaliana*. J. Biol. Chem. 276, 4338–4343. 10.1074/jbc.M007447200 11042215

[B36] Lin J.-S. Huang X.-X. Li Q. Cao Y. Bao Y. Meng X.-F.. (2016). UDP-glycosyltransferase 72B1 catalyzes the glucose conjugation of monolignols and is essential for the normal cell wall lignification in *Arabidopsis thaliana*. Plant J. 88, 26–42. 10.1111/tpj.13229 27273756

[B37] Liu C.-J. (2012). Deciphering the enigma of lignification: precursor transport, oxidation, and the topochemistry of lignin assembly. Mol. Plant 5, 304–317. 10.1093/mp/ssr121 22307199

[B38] Loughrin J. H. Hamilton-Kemp T. R. Burton H. R. Andersen R. A. Hildebrand D. F. (1992). Glycosidically bound volatile components of *Nicotiana sylvestris* and *N. Suaveolens* flowers. Phytochemistry 31, 1537–1540. 10.1016/0031-9422(92)83101-4

[B39] Louveau T. Leitao C. Green S. Hamiaux C. van der Rest B. Dechy-Cabaret O.. (2011). Predicting the substrate specificity of a glycosyltransferase implicated in the production of phenolic volatiles in tomato fruit. FEBS J. 278, 390–400. 10.1111/j.1742-4658.2010.07962.x 21166996

[B40] Lynch J. H. Orlova I. Zhao C. Guo L. Jaini R. Maeda H.. (2017). Multifaceted plant reponses to circumvent Phe hyperaccumulation by downregulation of flux through the shikimate pathway and by vacuolar Phe sequestration. Plant J. [Epub ahead of print]. 10.1111/tpj.13730 28977710

[B41] Miao Y.-C. Liu C.-J. (2010). ATP-binding cassette-like transporters are involved in the transport of lignin precursors across plasma and vacuolar membranes. Proc. Natl. Acad. Sci. U.S.A. 107, 22728–22733. 10.1073/pnas.1007747108 21149736 PMC3012465

[B42] Moerkercke A. V. Schauvinhold I. Pichersky E. Haring M. A. Schuurink R. C. (2009). A plant thiolase involved in benzoic acid biosynthesis and volatile benzenoid production. Plant J. 60, 292–302. 10.1111/j.1365-313X.2009.03953.x 19659733

[B43] Moon J.-H. Watanabe N. Ijima Y. Yagi A. Sakata K. (1996). cis- and trans-Linalool 3, 7-oxides and methyl salicylate glycosides and (Z)-3-hexenyl β-D-glucopyranoside as aroma precursors from tea leaves for oolong tea. Biosci. Biotechnol. Biochem. 60, 1815–1819. 10.1271/bbb.60.1815 8987857

[B44] Muhlemann J. K. Klempien A. Dudareva N. (2014). Floral volatiles: from biosynthesis to function. Plant Cell Environ. 37, 1936–1949. 10.1111/pce.12314 24588567

[B45] Oba K. Conn E. E. Canut H. Boudet A. M. (1981). Subcellular localization of 2-(β-d-glucosyloxy)-cinnamic acids and the related β-glucosidase in leaves of *Melilotus alba* Desr. Plant Physiol. 68, 1359–1363. 10.1104/pp.68.6.1359 16662108 PMC426103

[B46] Oka N. Ohishi H. Hatano T. Hornberger M. Sakata K. Watanabe N. (1999). Aroma evolution during flower opening in *Rosa damascena* mill. Zeitschrift fur Naturforschung C J. Biosci. 54, 889–895. 10.1515/znc-1999-1106

[B47] Ortiz-Serrano P. Gil J. V. (2010). Quantitative comparison of free and bound volatiles of two commercial tomato cultivars (*Solanum lycopersicum* L.) during ripening. J. Agric. Food Chem. 58, 1106–1114. 10.1021/jf903366r 20014855

[B48] Oyama-Okubo N. Ando T. Watanabe N. Marchesi E. Uchida K. Nakayama M. (2005). Emission mechanism of floral scent in *Petunia axillaris*. Biosci. Biotechnol. Biochem. 69, 773–777. 10.1271/bbb.69.773 15849416

[B49] Pan H. Zhou R. Louie G. V. Muhlemann J. K. Bomati E. K. Bowman M. E.. (2014). Structural studies of cinnamoyl-CoA reductase and cinnamyl-alcohol dehydrogenase, key enzymes of monolignol biosynthesis. Plant Cell 26, 3709–3727. 10.1105/tpc.114.127399 25217505 PMC4213152

[B50] Pérez-Díaz R. Ryngajllo M. Pérez-Díaz J. Pe-a-Cortés H. Casaretto J. González-Villanueva E.. (2014). VvMATE1 and VvMATE2 encode putative proanthocyanidin transporters expressed during berry development in *Vitis vinifera* L. Plant Cell Rep. 33, 1147–1159. 10.1007/s00299-014-1604-9 24700246

[B51] Picone J. Clery R. Watanabe N. MacTavish H. Turnbull C. N. (2004). Rhythmic emission of floral volatiles from *Rosa damascena* semperflorens cv. ‘Quatre Saisons’. Planta 219, 468–478. 10.1007/s00425-004-1250-5 15054660

[B52] Rambla J. L. Tikunov Y. M. Monforte A. J. Bovy A. G. Granell A. (2014). The expanded tomato fruit volatile landscape. J. Exp. Bot. 65, 4613–4623. 10.1093/jxb/eru128 24692651

[B53] Ravid J. Spitzer-Rimon B. Takebayashi Y. Seo M. Cna'ani A. Aravena-Calvo J.. (2017). GA as a regulatory link between the showy floral traits color and scent. New Phytol. 215, 411–422. 10.1111/nph.14504 28262954

[B54] Robert S. Zouhar J. Carter C. Raikhel N. (2007). Isolation of intact vacuoles from Arabidopsis rosette leaf-derived protoplasts. Nat. Protoc. 2, 259–262. 10.1038/nprot.2007.26 17406583

[B55] Sakai M. Tomita S. Hirata H. Asai T. Dohra H. Hara M.. (2008). Purification and characterization of β-glucosidase involved in the emission of 2-phenylethanol from rose flowers. Biosci. Biotechnol. Biochem. 72, 219–221. 10.1271/bbb.70404 18175907

[B56] Schuurink R. C. Haring M. A. Clark D. G. (2006). Regulation of volatile benzenoid biosynthesis in petunia flowers. Trends Plant Sci. 11, 20–25. 10.1016/j.tplants.2005.09.009 16226052

[B57] Sheehan H. Hermann K. Kuhlemeier C. (2012). Color and scent: how single genes influence pollinator attraction. Cold Spring Harbor Symp. Quant. Biol. 77, 117–133. 10.1101/sqb.2013.77.014712 23467550

[B58] Spitzer-Rimon B. Farhi M. Albo B. Cna'ani A. Ben Zvi M. M. Masci T.. (2012). The R2R3-MYB-like regulatory factor EOBI, acting downstream of EOBII, regulates scent production by activating ODO1 and structural scent-related genes in petunia. Plant Cell 24, 5089–5105. 10.1105/tpc.112.105247 23275577 PMC3556977

[B59] Spitzer-Rimon B. Marhevka E. Barkai O. Marton I. Edelbaum O. Masci T.. (2010). EOBII, a gene encoding a flower-specific regulator of phenylpropanoid volatiles' biosynthesis in petunia. Plant Cell 22, 1961–1976. 10.1105/tpc.109.067280 20543029 PMC2910970

[B60] Szeja W. Grynkiewicz G. Rusin A. (2017). Isoflavones, their glycosides and glycoconjugates. Synthesis and biological activity. Curr. Org. Chem. 21, 218–235. 10.2174/1385272820666160928120822 28553156 PMC5427819

[B61] Tikunov Y. M. de Vos R. C. H. González Paramás A. M. Hall R. D. Bovy A. G. (2010). A role for differential glycoconjugation in the emission of phenylpropanoid volatiles from tomato fruit discovered using a metabolic data fusion approach. Plant Physiol. 152, 55–70. 10.1104/pp.109.146670 19889876 PMC2799346

[B62] Tikunov Y. M. Molthoff J. de Vos R. C. H. Beekwilder J. van Houwelingen A. van der Hooft J. J. J.. (2013). Non-smoky Glycosyltransferase1 prevents the release of smoky aroma from tomato fruit. Plant Cell 25, 3067–3078. 10.1105/tpc.113.114231 23956261 PMC3784599

[B63] Tiwari P. Sangwan R. S. Sangwan N. S. (2016). Plant secondary metabolism linked glycosyltransferases: an update on expanding knowledge and scopes. Biotechnol. Adv. 34, 714–739. 10.1016/j.biotechadv.2016.03.006 27131396

[B64] Vainstein A. Sharon R. (1993). Biogenesis of petunia and carnation corolla chloroplasts: changes in the abundance of nuclear and plastid-encoded photosynthesis-specific gene products during flower development. Physiol. Plant. 89, 192–198. 10.1111/j.1399-3054.1993.tb01805.x

[B65] Van Moerkercke A. Galván-Ampudia C. S. Verdonk J. C. Haring M. A. Schuurink R. C. (2012). Regulators of floral fragrance production and their target genes in petunia are not exclusively active in the epidermal cells of petals. J. Exp. Bot. 63, 3157–3171. 10.1093/jxb/ers034 22345641 PMC3350925

[B66] Vandenbussche M. Chambrier P. Rodrigues Bento S. Morel P. (2016). Petunia, your next supermodel? Front. Plant Sci. 7:72. 10.3389/fpls.2016.00072 26870078 PMC4735711

[B67] Vishnevetsky M. Ovadis M. Zuker A. Vainstein A. (1999). Molecular mechanisms underlying carotenogenesis in the chromoplast: multilevel regulation of carotenoid-associated genes. Plant J. 20, 423–431. 10.1046/j.1365-313x.1999.00615.x 10607295

[B68] Watanabe S. Hashimoto I. Hayashi K. Yagi K. Asai T. Knapp H.. (2001). Isolation and identification of 2-phenylethyl disaccharide glycosides and mono glycosides from rose flowers, and their potential role in scent formation. Biosci. Biotechnol. Biochem. 65, 442–445. 10.1271/bbb.65.442 11302185

[B69] Wei S. Marton I. Dekel M. Shalitin D. Lewinsohn E. Bravdo B.-A. (2004). Manipulating volatile emission in tobacco leaves by expressing *Aspergillus niger* β-glucosidase in different subcellular compartments. Plant Biotechnol. J. 2, 341–350. 10.1111/j.1467-7652.2004.00077.x 17134395

[B70] Widhalm J. R. Gutensohn M. Yoo H. Adebesin F. Qian Y. Guo L.. (2015). Identification of a plastidial phenylalanine exporter that influences flux distribution through the phenylalanine biosynthetic network. Nat. Commun. 6:8142. 10.1038/ncomms9142 26356302 PMC4647861

[B71] Yilmaztekin M. Kocabey N. Hayaloglu A. A. (2015). Effect of maceration time on free and bound volatiles of red wines from cv. Karaoglan (*Vitis vinifera* L.) grapes grown in Arapgir, Turkey. J. Food Sci. 80, C556–C563. 10.1111/1750-3841.12767 25677953

[B72] Yon F. Kessler D. Joo Y. Cortés Llorca L. Kim S.-G. Baldwin I. T. (2017). Fitness consequences of altering floral circadian oscillations for *Nicotiana attenuata*. J. Integr. Plant Biol. 59, 180–189. 10.1111/jipb.12511 27957809

[B73] Zhang C. Hicks G. R. Raikhel N. V. (2014). Plant vacuole morphology and vacuolar trafficking. Front. Plant Sci. 5:476. 10.3389/fpls.2014.00476 25309565 PMC4173805

[B74] Zhang Z.-Z. Li Y.-B. Qi L. Wan X.-C. (2006). antifungal activities of major tea leaf volatile constituents toward *Colletorichum camelliae* Massea. J. Agric. Food Chem. 54, 3936–3940. 10.1021/jf060017m 16719518

[B75] Zhao C. L. Chen Z. J. Bai X. S. Ding C. Long T. J. Wei F. G.. (2014). Structure–activity relationships of anthocyanidin glycosylation. Mol. Divers. 18, 687–700. 10.1007/s11030-014-9520-z 24792223

[B76] Zhou Y. Dong F. Kunimasa A. Zhang Y. Cheng S. Lu J.. (2014). Occurrence of glycosidically conjugated 1-phenylethanol and its hydrolase β-primeverosidase in tea (*Camellia sinensis*) flowers. J. Agric. Food Chem. 62, 8042–8050. 10.1021/jf5022658 25065942

