# Supplementary material for: Phenylpropanoid Scent Compounds in Petunia x hybrida Are Glycosylated and Accumulate in Vacuoles
Source: Front Plant Sci. 2017 Nov 3;8:1898. doi: 10.3389/fpls.2017.01898 (PMC5675896; doi:10.3389/fpls.2017.01898)
Supplement: Supplementary file 1 [file DataSheet1.DOCX]

Supplementary Material

**Phenylpropanoid Scent Compounds in *Petunia* x *hybrida* Are Glycosylated and Accumulate in Vacuoles**

Alon Cna'ani^1^, Reut Shavit^2^, Jasmin Ravid^1^, Javiera Aravena-Calvo^1^, Oded Skaliter^1^, Tania Masci^1^ and Alexander Vainstein^1,*^

**^*^ Correspondence:**

Alexander Vainstein

alexander.vainstein@mail.huji.ac.il

## Supplementary Figures

**Supplementary Figure 1.** **Headspace emitted volatiles of flowers of petunia lines Blue Ray, W115 (MD) and P720.** (A) Dynamic headspace analyses of total emitted volatiles followed by GC–MS performed for 24 h on corollas of Blue Ray, W115 (MD) and P720 petunias. (B–D) Analyses of individual emitted volatiles are presented for (B) Blue Ray, (C) P720 and (D) W115 (MD) petunias. Columns represent mean values of two to five independent experiments. SEs are indicated by vertical lines.

**Supplementary Figure 2. Volatile emission patterns during flower development.** Dynamic headspace analyses of individual emitted volatiles followed by GC–MS were performed for 24 h at different developmental stages on corollas of petunia P720. (A–E) Levels of emission of (A) benzyl alcohol, (B) phenylethyl alcohol, (C) eugenol, (D) isoeugenol and (E) vanillin. Columns represent mean values of three to five independent experiments. SEs are indicated by vertical lines.

**Supplementary Figure 3**. **Volatile emission patterns throughout the day.** Dynamic headspace analyses of individual emitted volatiles followed by GC–MS performed for 24 h at 4-h intervals on corollas of petunia P720. (A–E) Levels of emission of (A) benzyl alcohol, (B) phenylethyl alcohol, (C) eugenol, (D) isoeugenol and (E) vanillin. Columns represent mean values of three to five independent experiments. SEs are indicated by vertical lines.

**Supplementary Figure 4**. **Efficiency of phenylacetaldehyde labeling following feeding with a stable isotope of ^2^H_5_-Phenylalanine.** Detached petunia corollas (1 DPA, 1500 h) were fed for 20 min with ^2^H_5_-Phe and metabolites were immediately extracted and analyzed by GC–MS. Columns represent mean values of three independent experiments. SEs are indicated by vertical lines. Significance of differences between treatments was calculated by Student’s t-test, **P* ≤ 0.05.

**Supplementary Figure 5. Mass spectrum of scent compounds extracted from petunia P720 flowers labeled with ^2^H_5_-Phe.** (A) Benzyl alcohol. (B) Phenylethyl alcohol. (C) Vanillin. (D) Isoeugenol. Presented are the mass spectra of I – authentic standard, II – non-labeled compound, III – isotopomer and IV – where available, metabolic pathway originating from the labeled precursor phenylalanine (^2^H_5_-Phe) to the labeled product.

**Supplementary Figure 6. Levels of aglycones in petunia P720 petals infiltrated with pBINPLUS-35S:*AnBGL1vac* + pRCS2-35S:*GFP* and pRCS2-35S:*GFP* (control).** Presented are the levels of the following aglycones: **(A)** benzyl alcohol, **(B)** phenylethyl alcohol and **(C)** vanillin. Columns represent the mean values of four to six independent experiments. SEs are indicated by vertical lines. Significance of differences between treatments was calculated by Student’s t-test, **P* ≤ 0.05. n.d – not detected.
