# Supplementary figures and images for: Phenylpropanoid Scent Compounds in Petunia x hybrida Are Glycosylated and Accumulate in Vacuoles
Source: Front Plant Sci. 2017 Nov 3;8:1898. doi: 10.3389/fpls.2017.01898 (PMC5675896; doi:10.3389/fpls.2017.01898)

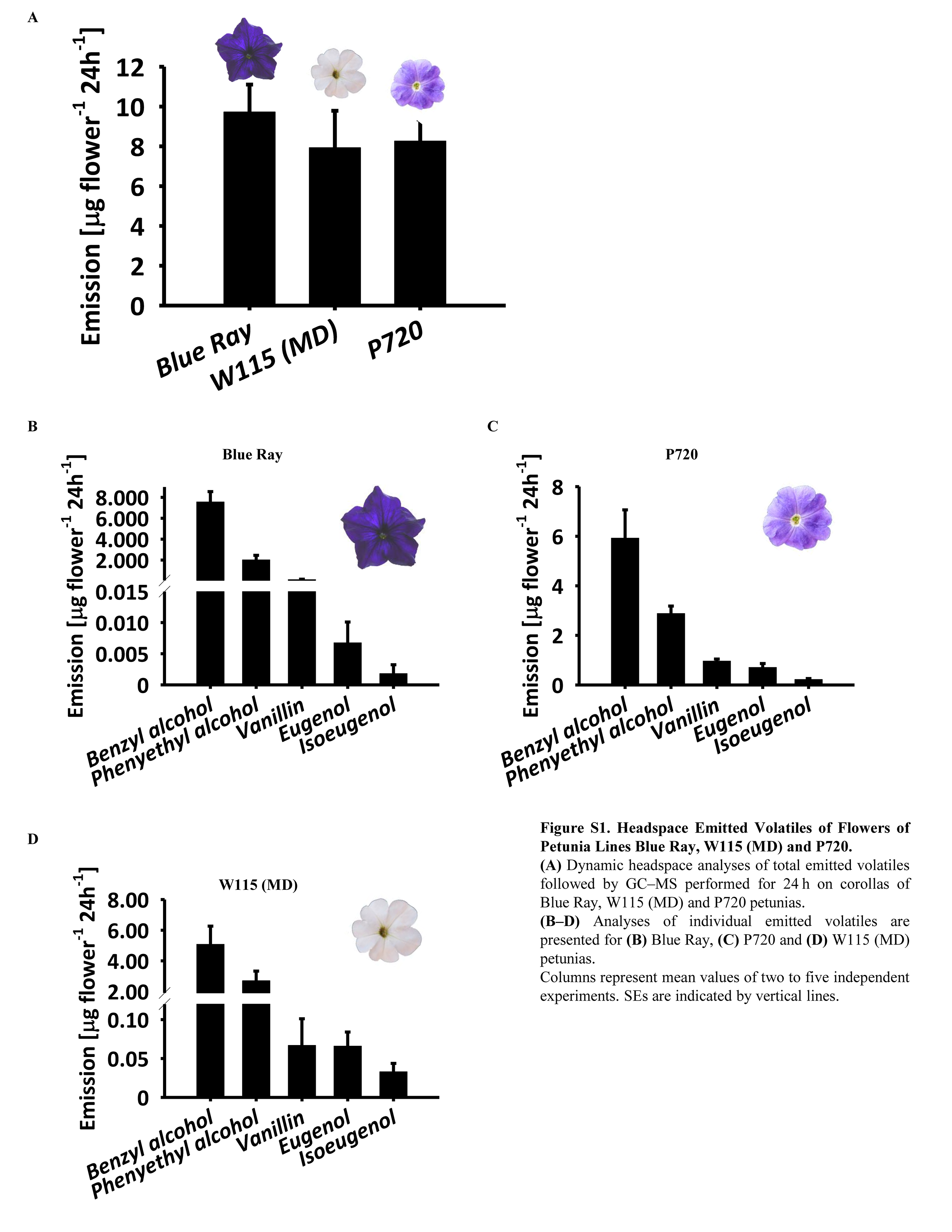

Supplement: Supplementary file 2 [file Image1.TIF]

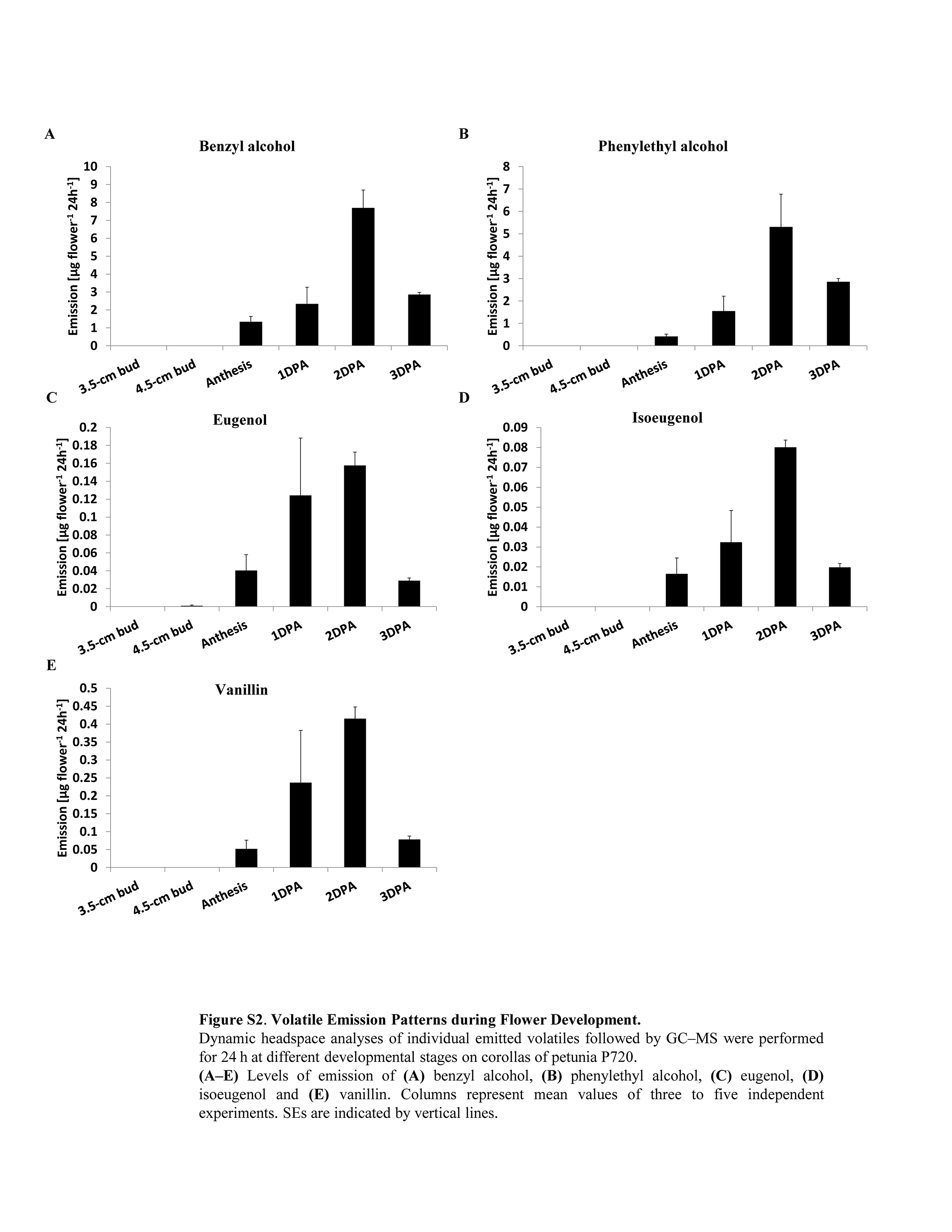

Supplement: Supplementary file 3 [file Image2.TIF]

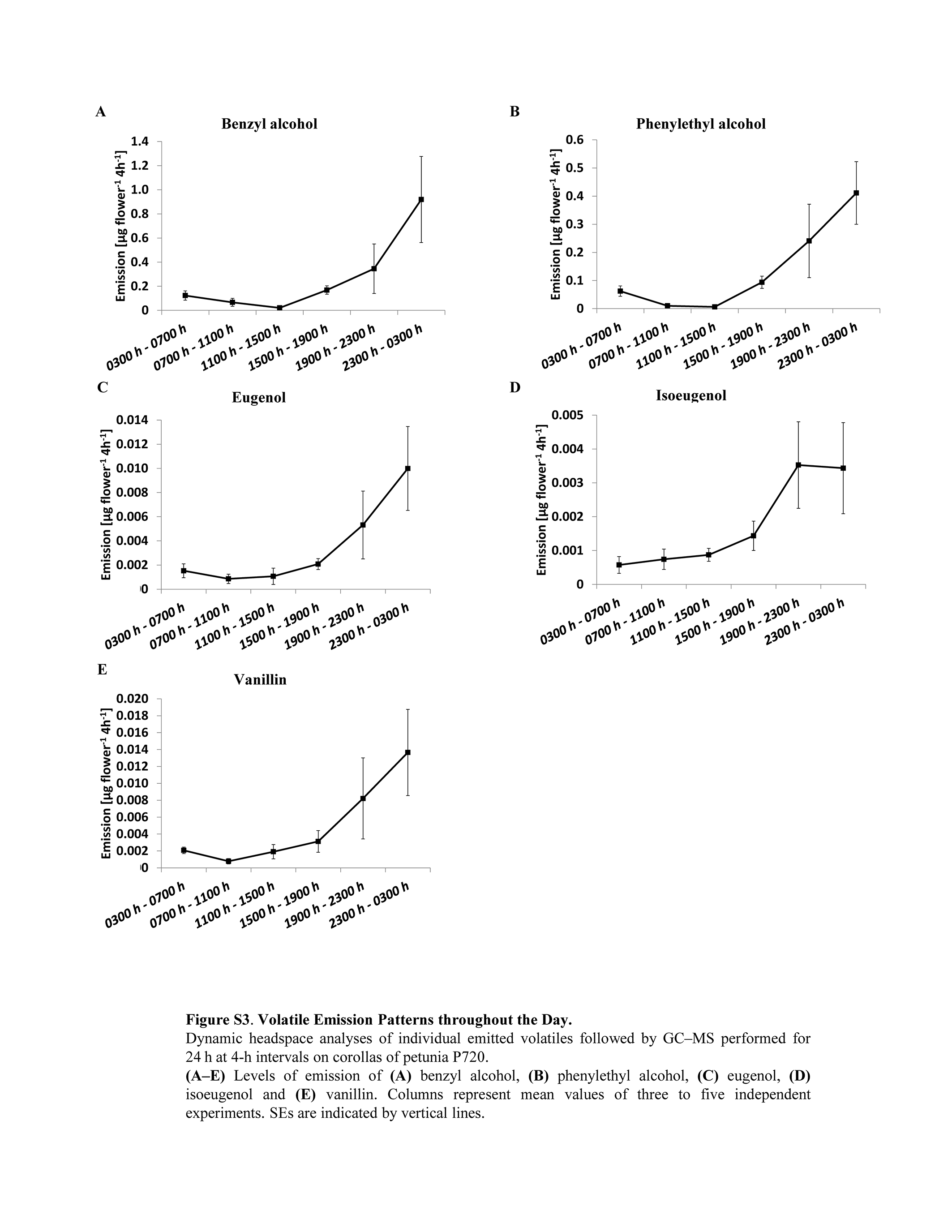

Supplement: Supplementary file 4 [file Image3.TIF]

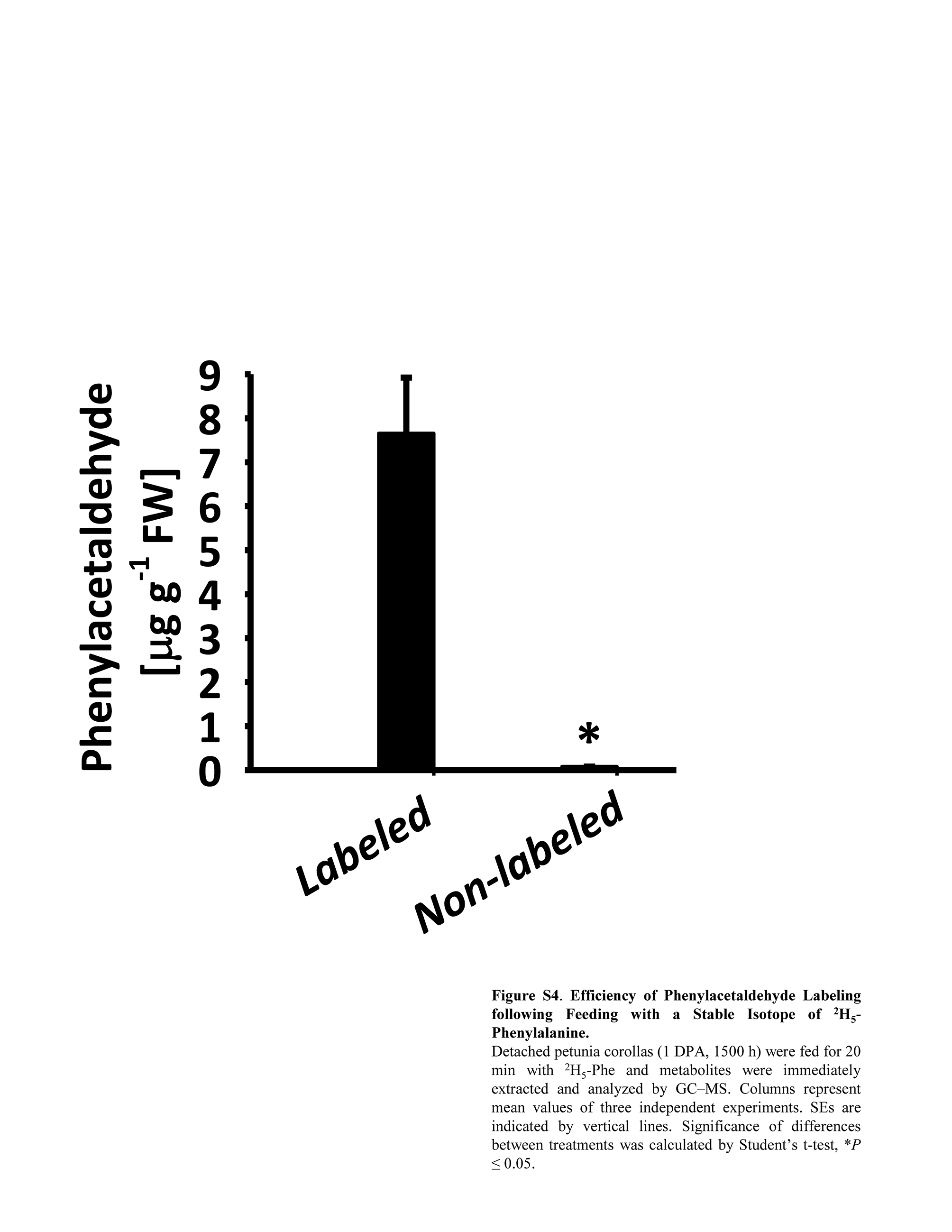

Supplement: Supplementary file 5 [file Image4.TIF]

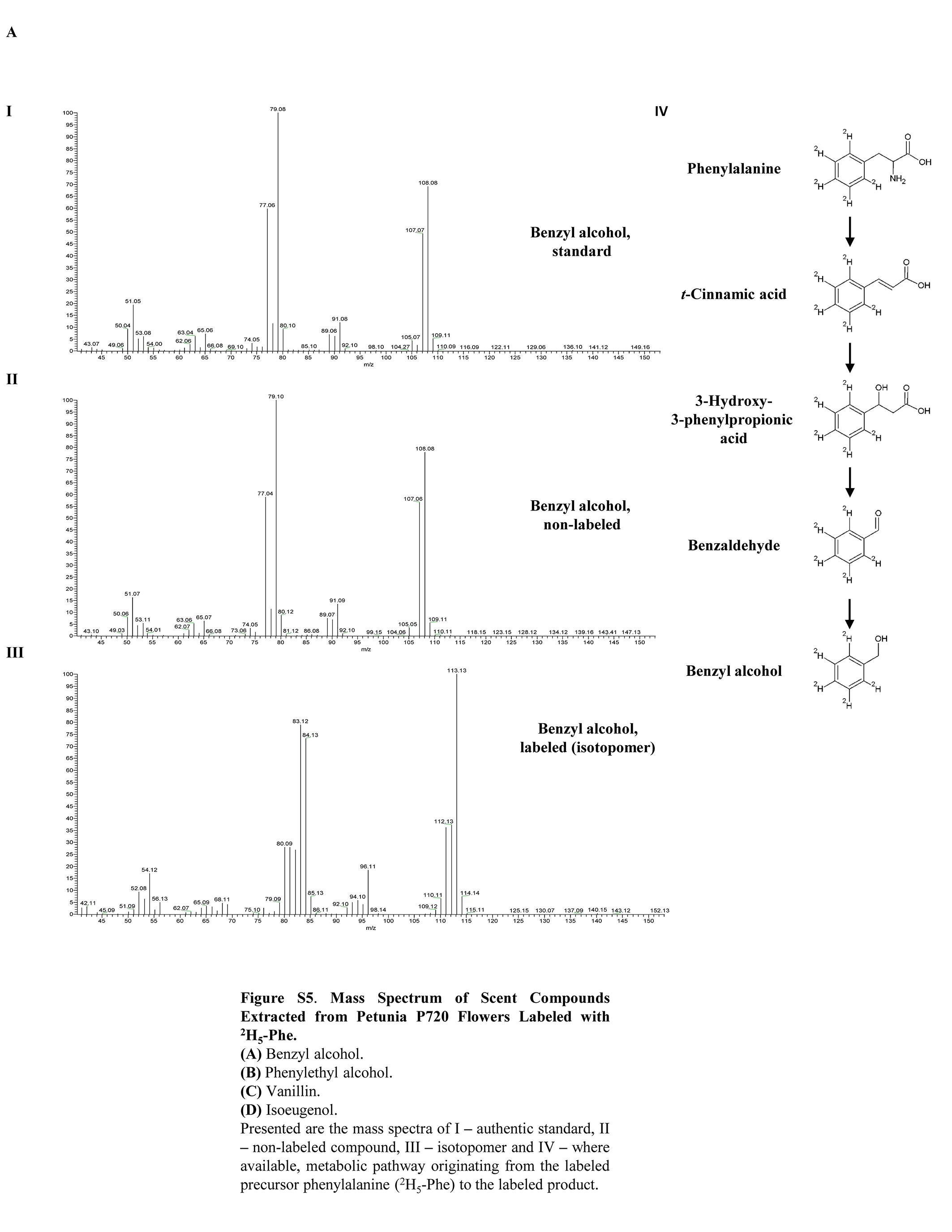

Supplement: Supplementary file 6 [file Image5A.TIF]

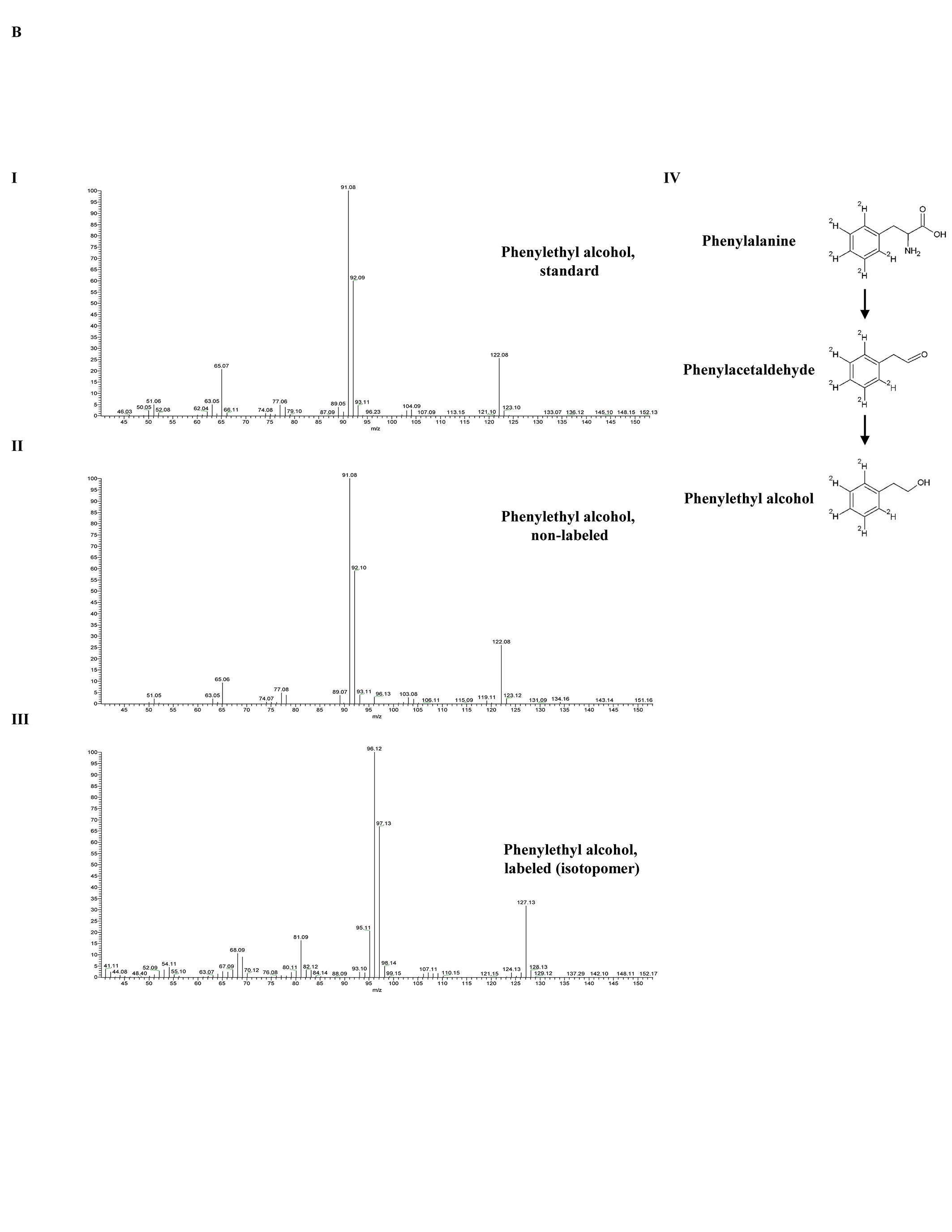

Supplement: Supplementary file 7 [file Image5B.TIF]

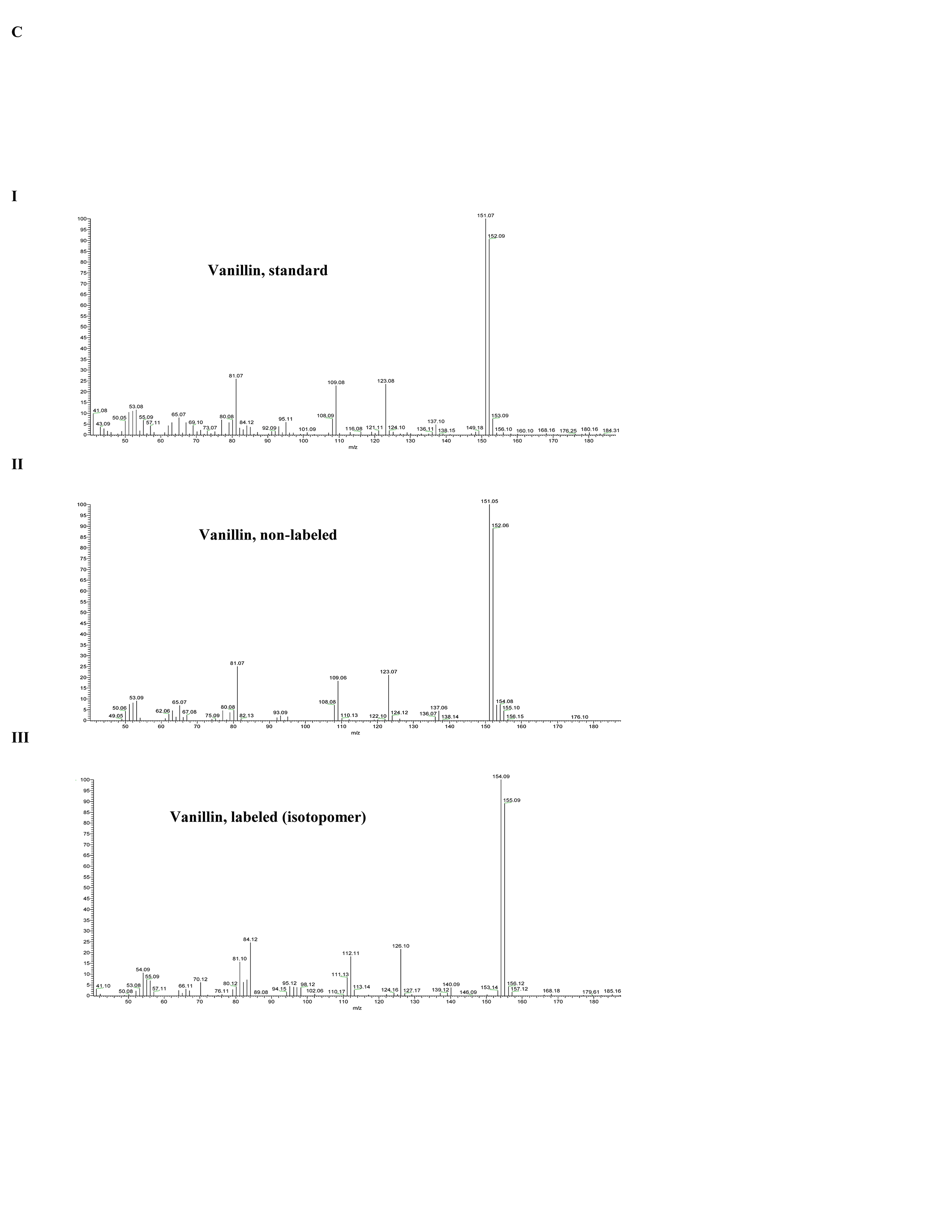

Supplement: Supplementary file 8 [file Image5C.TIF]

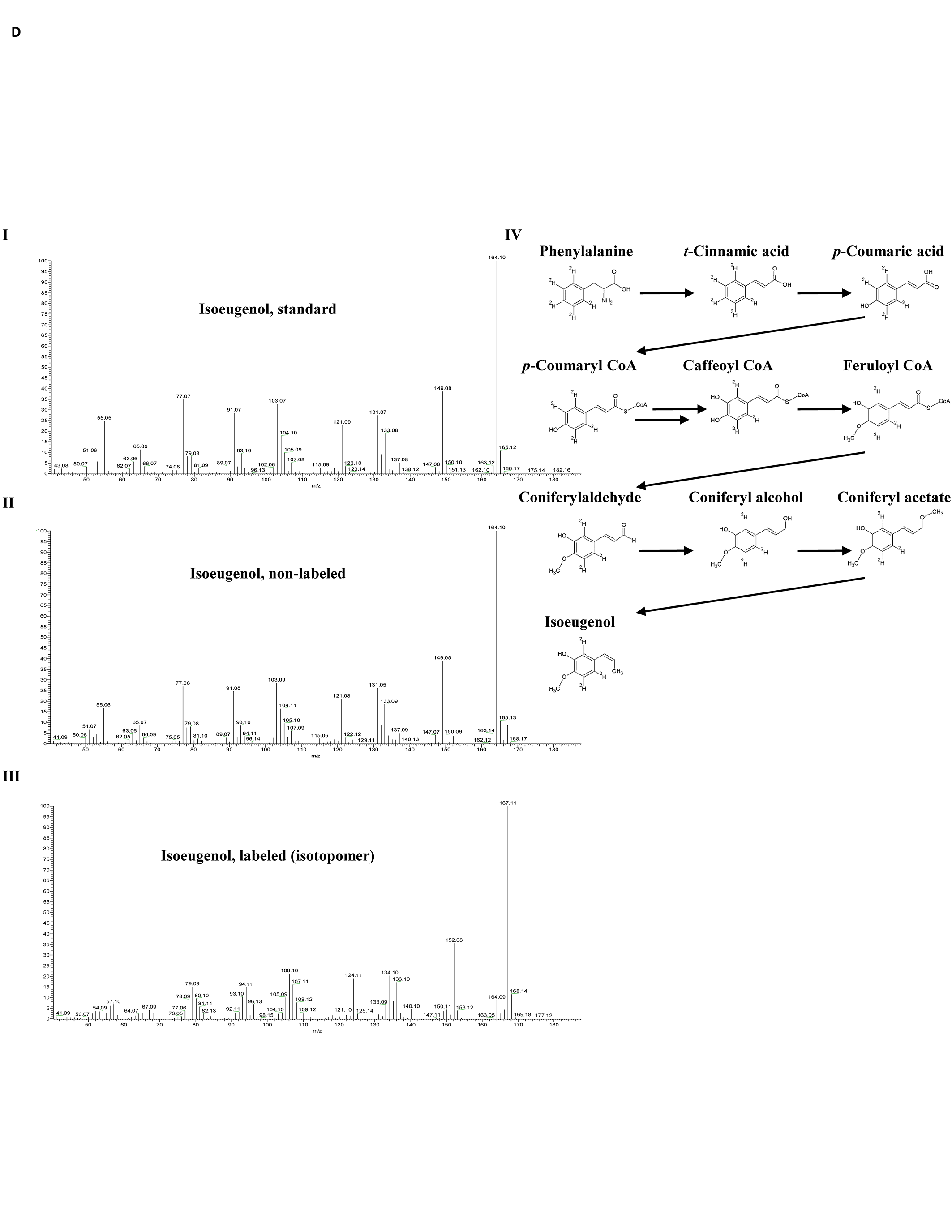

Supplement: Supplementary file 9 [file Image5D.TIF]

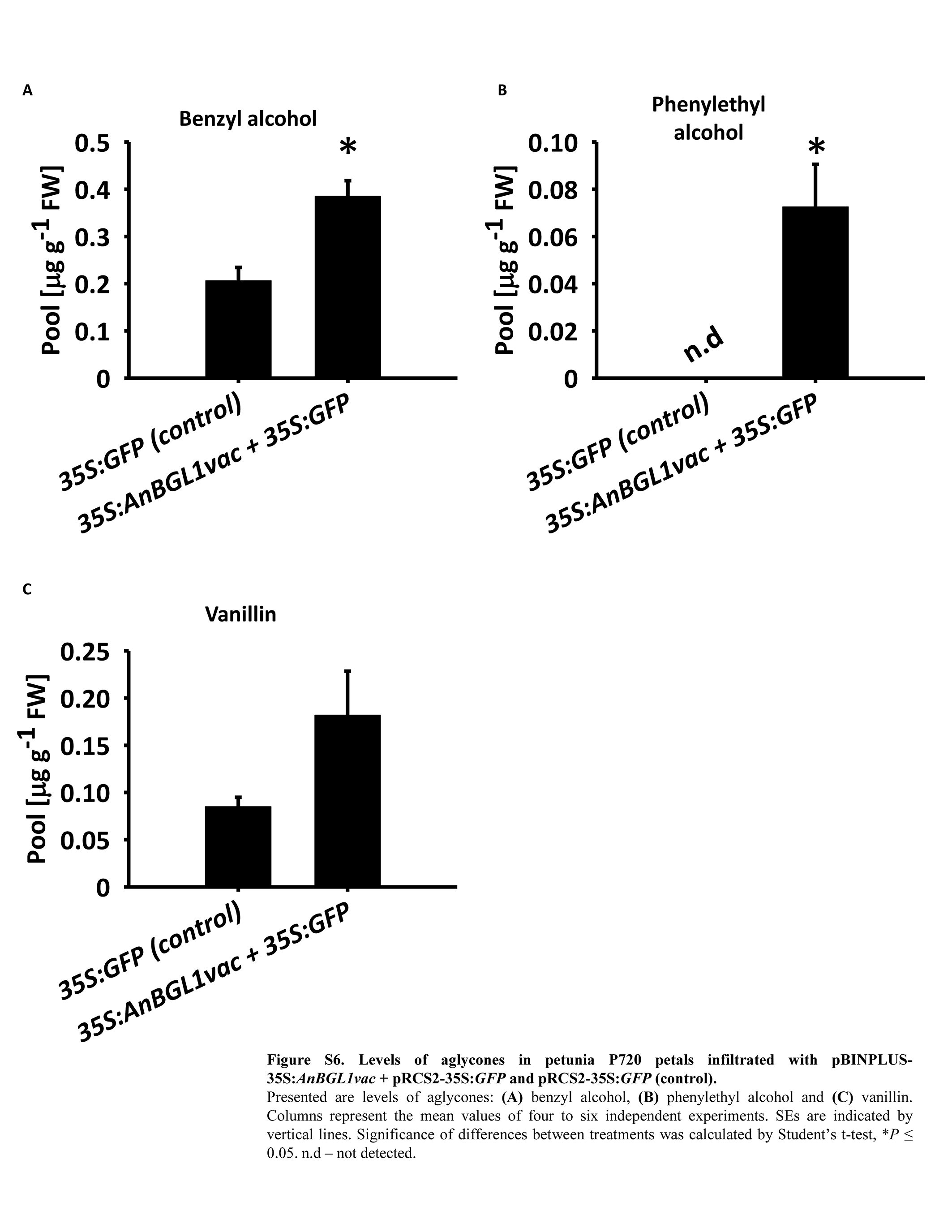

Supplement: Supplementary file 10 [file Image6.TIF]
